# The oncogenic axis YAP/MYC/EZH2 impairs PTEN tumor suppression activity enhancing lung tumorigenicity

**DOI:** 10.1038/s41420-024-02216-8

**Published:** 2024-10-25

**Authors:** Federica Lo Sardo, Chiara Turco, Beatrice Messina, Andrea Sacconi, Francesca Romana Auciello, Claudio Pulito, Sabrina Strano, Sima Lev, Giovanni Blandino

**Affiliations:** 1 Translational Oncology Research Unit, Department of Research, Diagnosis and Innovative Technologies, IRCCS Regina Elena National Cancer Institute, Rome, Italy; 2 Clinical Trial Center, Biostatistics and Bioinformatics Unit, Department of Research, Diagnosis and Innovative Technologies, IRCCS Regina Elena National Cancer Institute, Rome, Italy; 3 SAFU Laboratory, Department of Research, Advanced Diagnostic, and Technological Innovation, IRCCS, Regina Elena National Cancer Institute, Rome, Italy; 4 Molecular Cell Biology Department, Weizmann Institute of Science, Rehovot, Israel

**Keywords:** Non-small-cell lung cancer, Gene silencing

## Abstract

The tumor suppressor PTEN (phosphatase and tensin homolog deleted in chromosome 10) is genetically deleted or downregulated in many cancer types. Loss of PTEN protein expression is frequently found in lung cancer while genetic alterations are less abundant. PTEN expression is regulated at multiple genetic and epigenetic levels and even partial reduction of its expression increases cancer occurrence. We show that YAP and TAZ cooperate with EZH2, and MYC to transcriptionally repress onco-suppressor genes, including PTEN, in non-small cell lung cancer (NSCLC) cells. YAP/TAZ-EZH2-MYC transcriptional regulators form a nuclear complex that represses PTEN transcription, while their combinatorial targeting restores PTEN expression, attenuates NSCLC cell growth, and prevents compensatory responses induced by single treatments. Datasets analysis of NSCLC patients revealed that PTEN expression is negatively correlated to YAP/TAZ, EZH2 and MYC and that low expression of PTEN is predictive of poor prognosis, especially at earlier stages of the disease. These findings highlight the repressive role of the YAP/TAZ-EZH2-MYC axis on tumor-suppressor genes and offer a potential therapeutic strategy for lung cancer patients with low PTEN levels.

## Introduction

Lung cancer is the leading cause of cancer-related mortality in the world. It is classified into two major subtypes exhibiting different origin tissue, histology and response to therapy: small cell lung cancer (SCLC) accounts for 10–15% of all lung cancers while non-small cell lung cancer (NSCLC) for 80–85% of cases (https://cancerstatisticscenter.cancer.org). NSCLC is further categorized into lung adenocarcinoma (LUAD), the most common subtype arising from secretory cells of distal airways, and lung squamous cell carcinoma (LUSC) that, together with large cell carcinoma (LCC), originates from more proximal airways [1–4]. The high mortality rate of lung cancer, despite its relatively low incidence rate, is frequently associated with late detection at advanced stages. Nevertheless, in recent years, the death rate is progressively declining due to powerful early detection, reduced smoking, and improved treatments using target- and immuno-therapy in addition to traditional chemo- and radio-therapy (https://cancerstatisticscenter.cancer.org) [4–6]. Yet, not all patients are eligible for target- and immuno-therapy, and even the eligible ones, frequently develop resistance over time. It is, therefore, important to identify potent targets and to define the underlying mechanisms of resistance to commonly used therapies.

PTEN is a key onco-suppressor, which negatively regulates the PI3K/AKT/mTOR oncogenic pathway, and is involved in DNA repair. PTEN downregulation is an early event in lung tumorigenesis and it correlates with poor overall survival, progression-free survival and disease-free survival [7]. Molecularly, the reduction of PTEN expression induces the aberrant activation of the PI3K/AKT/mTOR pathway which in turn controls a plethora of oncogenic events, including cell growth, stemness, proliferation and survival, protein synthesis, energy metabolism, motility and cellular architecture [8–12]. Moreover, PTEN loss or downregulation confers resistance to radiotherapy, chemotherapy, target therapy and immunotherapy in some settings [13–15]. According to the Cancer Genome Atlas (TCGA), *PTEN* genetic mutations in LUSC are found in ~15% of cases, but only in ~2–3% of the LUAD patients. The low frequency of PTEN genetic alteration in LUAD cannot explain the higher frequency of downregulation of PTEN protein, which was shown to be dependent on multiple genetic and epigenetic mechanisms as well as protein–protein interactions [8]. In the present work, we show that YAP and TAZ cooperate with EZH2 and MYC to transcriptionally repress PTEN in LUAD cells. These transcriptional regulators maintain low levels of PTEN.

YAP and TAZ are widely known as transcriptional co-activators of different oncogenes, mainly those who are related to cell cycle, stemness and epithelial–mesenchymal transition (EMT). Genes commonly activated by those proteins in different cancer types are known as the oncogenic YAP- and TAZ-mediated gene signature [16]. In this work, we focus on the role of YAP and TAZ as transcriptional repressors of onco-suppressor genes. We observed that pharmacological inhibition of EZH2 or MYC alone inefficiently upregulates PTEN expression, possibly through activation of oncogenic pathways including the YAP/TAZ signature. However, the concomitant inhibition of YAP/TAZ together with EZH2 and MYC robustly derepressed the expression of PTEN and other onco-suppressor genes and suppressed the hyperactivation of the YAP and TAZ signature. Our study identified a novel oncogenic axis that can be targeted in lung cancer and highlights the suppression activity of YAP/TAZ on PTEN transcription.

## Results

### YAP, PRC2 and MYC contribute to the transcriptional repression of tumor-suppressor genes in NSCLC

Previous studies showed that YAP and EZH2 synergize to repress the transcription of lineage-specific genes and onco-suppressor genes in NSCLC, Schwann cells, trophoblast and intestine, and that YY1 (Yin Yang 1) transcription factor may bridge YAP and EZH2 at the promoter of cell-cycle regulators [17–20]. MYC was also proposed to cooperate with EZH2 or YY1 to repress the transcription of onco-suppressor genes in cancer [21–23] while cooperating with YAP/TAZ mainly to enhance the transcription of oncogenes [24].

**Fig. 1:**
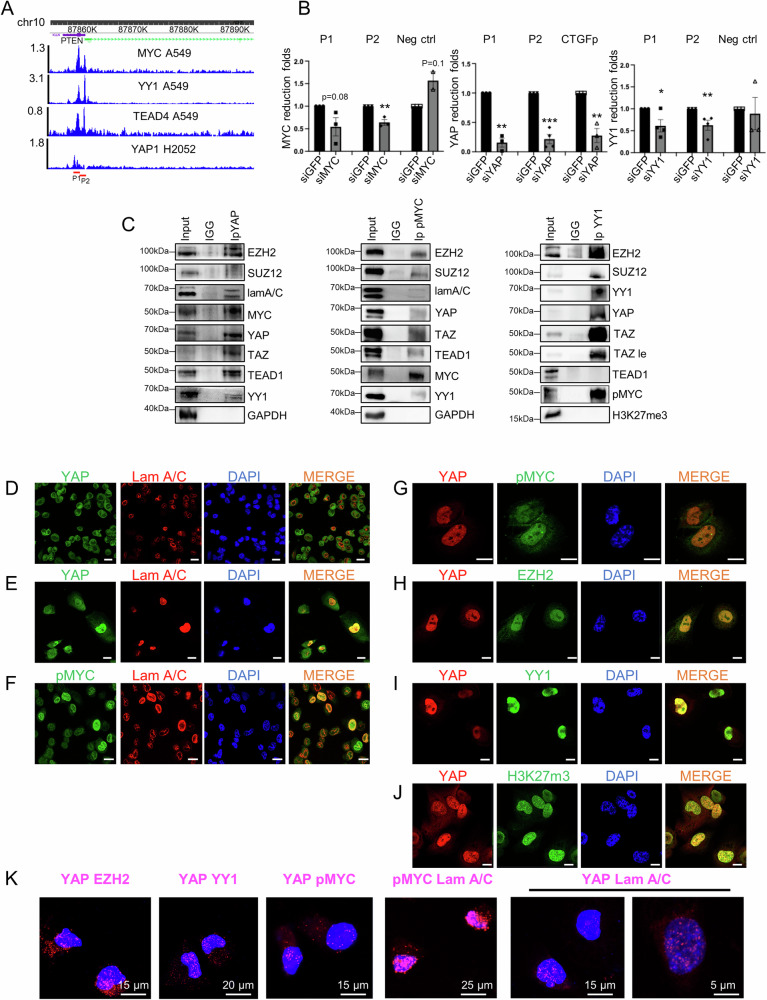
YAP, TEAD, PRC2 and MYC form a nuclear complex onto transcriptionally repressed onco-suppressor genes. **A** Chip-Seq tracks of YAP, MYC, TEAD4 and YY1 on the PTEN gene as obtained through the Cistrome Browser DB tool and displayed in the WashU Epigenome browser. MYC, YY1 and TEAD4 tracks were obtained in the A549 cell (https://genome.ucsc.edu/cgi-bin/hgTrackUi?db=hg19&g=wgEncodeHaibTfbs) [47]. YAP track was obtained in the NCI-H2052 cell line [48]. Red lines indicate the regions amplified for ChIp analysis. Chromosomal coordinates are relative to the Genome Reference Consortium Human Build 38 (hg38). **B** Chromatin immunoprecipitation analysis of the abundance of MYC, YAP and YY1 in H1299 cells depleted for MYC, YAP and YY1, respectively, compared to siGFP as control. Fold enrichment was calculated over IgG and then normalized to the siGFP control that was adjusted to 1. The experiments were performed in triplicate. An intronic region of the EZH2 locus was used as a negative control for MYC and YY1 binding, while CTGF promoter was used as a positive control for YAP binding. Two-tailed *t*-test analysis was applied to calculate the *P* values. *p < 0.05; **p < 0.01; ***p < 0.001. *p* and *n* values: PTENp1 siMYC vs siGFP *p* = 0.08, *n* = 3. PTENp2 siMYC vs siGFP *p* = 0.004, *n* = 3. neg ctrl siMYC vs siGFP *p* = 0.1, *n* = 2. CTGF siMYC vs siGFP *p* = 0.37, *n* = 2. **C** H1299 protein lysates were immunoprecipitated (IP) with anti-IgG, anti-YAP, anti-pMYC or anti-YY1 antibodies and then subjected to Western blot (WB) with the indicated antibodies (right side). 5–10% of total lysate was used as input control. Confocal analysis of YAP (green) and Lamin A/C (red) signals of H1299 cells seeded at higher density (**D**) or lower density condition (**E**). Magnification = ×63. Scale bar = 22 µm (**D**), 10 µm (**E**). **F** Confocal analysis of pMYC (green) and Lamin A/C (red) signals in H1299 cells seeded at high density. **G**–**J** Confocal analysis of the indicated proteins in H1299 cells seeded at low-density culture conditions. Magnification = ×63. Scale bar = 17 µm (**F**) and 10 µm (**G**–**J**). For each analysis, DAPI (blue) and merge are shown. **K** PLA for the analysis of the interaction between proteins indicated in panels. Magnification = ×63. Scale bar is indicated in panels.

Considering the oncogenic impact of PTEN loss on NSCLC tumorgenicity and its possible downregulation by transcriptional and post-transcriptional mechanisms, we examined its promoter region using the Cistrome Data Browser and found a clear co-localization of TEAD4, YY1 and MYC onto its promoter in the A549 NSCLC cell line (Fig. 1A). TEAD1-4 transcription factors are known to bind YAP/TAZ on both activated and repressed genes promoters. Therefore, despite the absence of YAP track in the Cistrome DB of A549 cells, we assume that YAP binds PTEN promoter since a YAP peak overlapping with TEAD4, YY1 and MYC is present in the mesothelioma cell line H2052 (Fig. 1A). The presence of YAP, MYC and YY1 on PTEN promoter was validated by chromatin immunoprecipitation (ChIP) in the H1299 NSCLC cell line (Fig. 1B). Furthermore, co-immunoprecipitation (Co-IP) and immunofluorescence (IF) experiments in H1299 cells revealed physical interaction and cellular co-localization of endogenous YAP with TEAD1, pMYC, EZH2, YY1 as well as with Lamin A/C, a structural protein associated with the inner nuclear membrane where the epigenetically repressed chromatin is clustered [25, 26] (Fig. 1C–J, S8). Interestingly, pMYC also co-immuno-precipitates, co-localizes and interacts in the nucleus with Lamin A/C (Fig. 1C, F, S9). We obtained similar patterns of YAP distribution and co-localization with Lamin A/C, pMYC, YY1 and H3K27me3 in H1975 cells (Fig. S1). Notably, H3K27me3 is enriched at the nuclear lamina (Fig. S1D) with a ring-like pattern, similar to YAP (Fig. S1A).

Proximity ligation assay (PLA) in H1299 confirmed in situ the interaction of YAP with EZH2, YY1, pMYC, and Lamin A/C as well as the interaction between pMYC and Lamin A/C (Fig. 1K).

By matching published lists of genes upregulated in YAP- or TAZ-depleted cells (GSE151200) [27], which are possibly target genes transcriptionally repressed by YAP or TAZ, with those that are bound by MYC (*ChIP*-*Atlas*) and either EZH2 [28] or H3K27me3 (GSE29611) in A549 cells, we obtained two distinct lists of genes (Table S1). These lists were obtained by applying pval < 0.05 and padj < 0.3. The heatmaps and Venn diagram shown in Fig. S2A, B show that TAZ/EZH2/MYC co-represses a higher number of genes (*N* = 165) compared to YAP/EZH2/MYC (*N* = 50 genes). Moreover, 20 genes are commonly repressed by both YAP and TAZ, indicating that almost half of YAP repressed genes (40%) are also repressed by TAZ. However, only 12% of genes repressed by TAZ are also repressed by YAP (Fig. S2A, B and Table S1). Genes set enrichment analysis (GSEA) of genes repressed by either YAP or TAZ and co-bound by MYC and EZH2/H3k27me3 highlights apical junction, apical surface, EMT, genes downregulated by KRAS activation and IL2-STAT5 signaling, as the most significant pathways affected by these proteins (Fig. S2C and Table S1, sheet 3). Collectively, these data suggest that the transcriptional repression mediated by YAP, TAZ, MYC and EZH2 may be a general mechanism, and supports the emerging notion that YAP and TAZ may have both overlapping and distinct functions not only in the transcriptional activation but also in the transcriptional repression of different subsets of genes [29].

### Oncogenic MYC cooperates with YAP and TAZ to repress transcription of PTEN

**Fig. 2:**
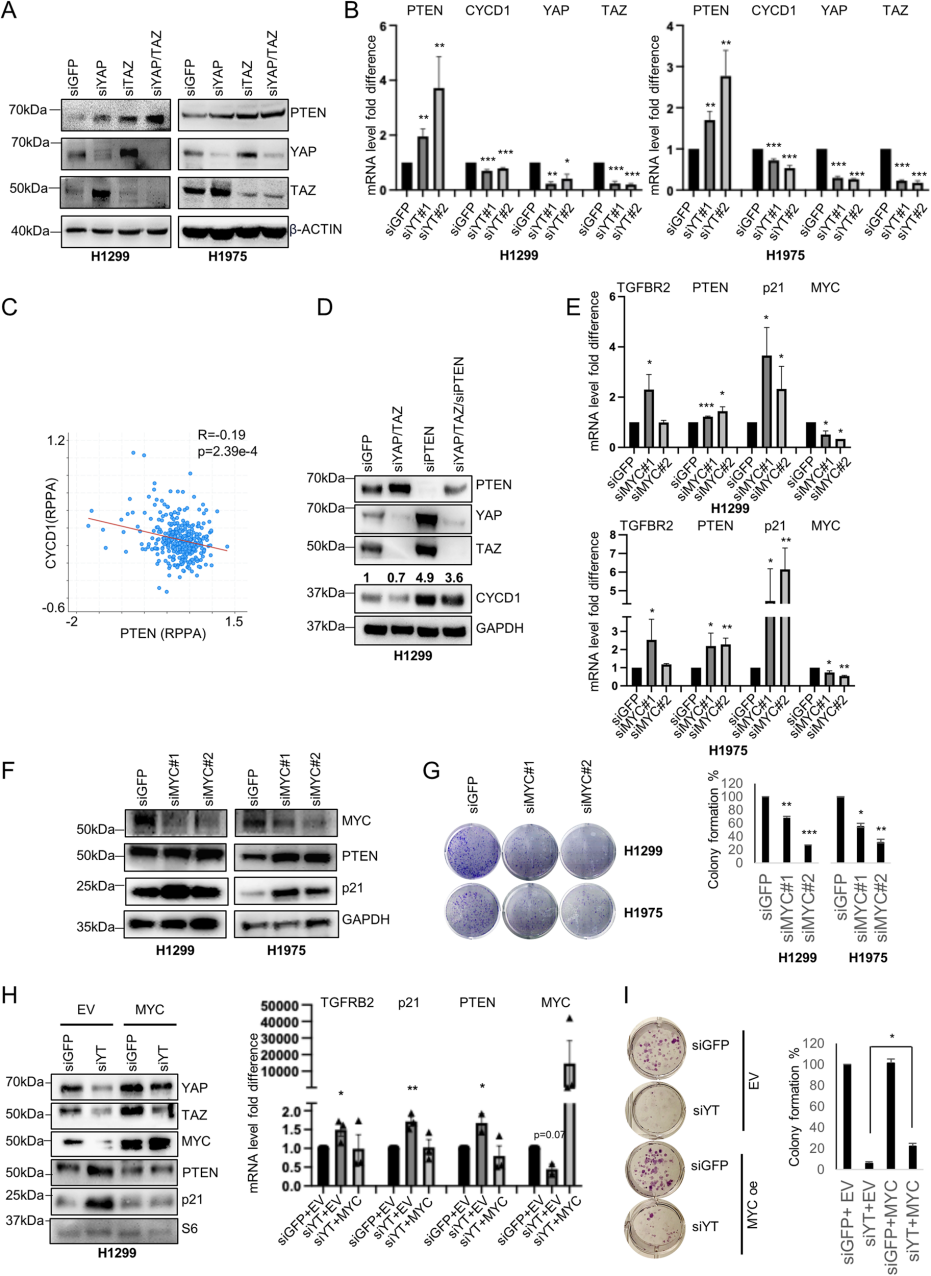
PTEN is transcriptionally repressed by YAP/TAZ, EZH2 and MYC. **A** WB of the indicated proteins in H1299 (left) and H1975 (right) upon knockdown (KD) of YAP, TAZ, or both (siYT), compared to siGFP as transfection control. β-actin was used as loading control. **B** Real-time quantification of the indicated transcripts in H1299 and H1975 cells upon YAP/TAZ KD with two alternative combinations of siRNAs. GAPDH was used for normalization. One-tailed *t*-test was applied to calculate the *p* value. The experiments were performed at least in triplicate. *p* and *n* values: H1299: PTEN siYT#1 vs siGFP *p* = 0.0018, *n* = 3. PTEN siYT#2 vs siGFP *p* = 0.015, *n* = 4. CYCD1 siYT#1 vs siGFP *p* = 0.0001, *n* = 3. CYCD1 siYT#2 vs siGFP *p* = 6.2e − 6, *n* = 3. YAP siYT#1 vs siGFP *p* = 0.0005, *n* = 3. YAP siYT#2 vs siGFP *p* = 0.02, *n* = 3. TAZ siYT#1 vs siGFP *p* = 8.4e − 7, *n* = 3. TAZ siYT#2 vs siGFP *p* = 2.6E − 10, *n* = 4. H1975: PTEN siYT#1 vs siGFP *p* = 0.001, *n* = 4. PTEN siYT#2 vs siGFP *p* = 0.005, *n* = 5. CYCD1 siYT#1 vs siGFP *p* = 4.8e − 8, *n* = 8. CYCD1 siYT#2 vs siGFP *p* = 3.9e − 9, *n* = 6. YAP siYT#1 vs siGFP *p* = 3e − 6, *n* = 3. YAP siYT#2 vs siGFP *p* = 1.2e − 7, *n* = 3. TAZ siYT#1 vs siGFP *p* = 1.8e − 7, *n* = 3. TAZ siYT#2 vs siGFP *p* = 5.9e − 6, *n* = 3. **C** Scatter plot analysis showing the correlation between PTEN and CYCD1 protein (*n* = 365) in lung adenocarcinoma patients as obtained from the cBioportal database by querying the lung adenocarcinoma TCGA, Firehose Legacy study. R indicates Pearson correlation. *p* value is derived from two-tailed *t*-test. **D** WB showing the abundance of the indicated proteins upon the indicated KD conditions. Numbers above CYCD1 blot indicate CYCD1 signal quantification normalized to GAPDH signal. Real-time quantification of transcripts (**E**) and WB analysis (**F**) of the indicated proteins in H1299 and H1975 cells at the indicated KD conditions. For each KD, at least two independent siRNAs were used. Each experiment was performed at least in triplicate. One-tailed *t*-test analysis was applied to calculate the *p* values. *p < 0.05; **p < 0.01; ***p < 0.001. *p* and *n* values: H1299: TGFBR2 siMYC#1 vs siGFP *p* = 0.03, *n* = 4. TGFBR2 siMYC#2 vs siGFP *p* = 0.4, *n* = 3. PTEN siMYC#1 vs siGFP *p* = 5e − 5, *n* = 4. PTEN siMYC#2 vs siGFP *p* = 0.01, *n* = 3. p21 siMYC#1 vs siGFP *p* = 0.02, *n* = 4. p21 siMYC#2 vs siGFP *p* = 0.03, *n* = 3. MYC siMYC#1 vs siGFP *p* = 0.02, *n* = 3. MYC siMYC#2 vs siGFP *p* = 0.04, *n* = 3. H1975: TGFBR2 siMYC#1 vs siGFP *p* = 0.05, *n* = 3. TGFBR2 siMYC#2 vs siGFP *p* = 0.003, *n* = 4. PTEN siMYC#1 vs siGFP *p* = 0.04, *n* = 4. PTEN siMYC#2 vs siGFP *p* = 0.001, *n* = 3. p21 siMYC#1 vs siGFP *p* = 0.02, *n* = 4. p21 siMYC#2 vs siGFP *p* = 0.0006, *n* = 4. MYC siMYC#1 vs siGFP *p* = 0.02, *n* = 3. MYC siMYC#2 vs siGFP *p* = 0.0002, *n* = 3. **G** Representative images (left) and quantification (right) of colony formation potential in H1299 and H1975 cells upon interference of MYC. Bar charts represent the mean and SEM of technical replicates from a representative experiment, which was repeated three times for each cell line. Two-tailed *t*-test analysis was applied to calculate the *p* values. H1299 siMYC#1 vs siGFP *p* = 0.0013, *n* = 3. siMYC#2 vs siGFP *p* = 0.0001, *n* = 3. H1975 siMYC#1 vs siGFP *p* = 0.01, *n* = 3. siMYC#2 vs siGFP *p* = 0.005, *n* = 3. *p < 0.05; **p < 0.01; ***p < 0.001. **H** WB and Real-time quantification of the indicated proteins and transcripts in H1299 upon YAP/TAZ KD with and without MYC overexpression. We used suboptimal concentration of siYAP/siTAZ to obtain a rescue upon MYC overexpression. S6 protein was used as loading control for WB. For real times, two-tailed *t*-test was applied to calculate the *p* value. *p* and *n* values for siYT + EV vs siGFP + EV: TGFBR2 *p* = 0.01, *n* = 3. p21 *p* = 0.0009, *n* = 3. PTEN *p* = 0.01, *n* = 43 MYC *p* = 0.07, *n* = 3. **I** Representative images (left) and quantification (right) of colony formation potential in H1299 and H1975 cells upon KD of YAP and TAZ with or without MYC overexpression. Bar charts represent the mean and SEM of technical replicates from a representative experiment, which was repeated three times. Two-tailed *t*-test analysis was applied to calculate the *p* values. siYT + EV vs siYT + MYC *p* = 0.05, *n* = 3.

We have noticed that PTEN was not present in the heatmaps of Fig. S2A and Table S1, possibly because the analysis included upregulated genes in response to single KD of either YAP or TAZ. Indeed, when YAP and TAZ were concomitantly depleted in two NSCLC cell lines, H1299 and H1975, a strong upregulation of PTEN was observed, concurrent with a decrease in CYCD1 (cyclin D1) transcript, a cell-cycle regulator that is negatively modulated by PTEN (Fig. 2A, B, S10A) [30]. Importantly, the expression of CYCD1 and PTEN are anti-correlated in NSCLC patients (Fig. 2C). KD of PTEN robustly upregulated CYCD1 and rescued CYCD1 levels in siYAP/TAZ double KD cells (Fig. 2D, S10B). Conversely, PTEN overexpression reduced CYCD1 levels and increased the fraction of cells at the G1/S transition in two other NSCLC cell lines, H1975 and H358, with no evident effects in H1299 cells (Figs. S2D, E, S13B) [30].

To evaluate the impact of MYC on PTEN expression, we downregulated its expression by siRNA in H1299 and H1975 cells, and observed a significant upregulation of PTEN only in H1975 cells (Fig. 2E, F, S10C). However, colony formation was inhibited in both cell lines upon MYC depletion (Fig. 2G), suggesting that MYC elicits its oncogenic function through several mechanisms beyond PTEN downregulation. Upon MYC depletion, we also observed a cell line-specific de-repression of TGFBR2 and p21, previously shown to be repressed by YAP, TAZ and EZH2, implying a similar repression mechanism for those genes (Fig. 2E, F, S10C) [18]. Although depletion of MYC alone had not affect PTEN expression in H1299 cells, we found that MYC overexpression in YAP/TAZ depleted H1299 cells could rescue PTEN, p21 and TGFBR2 repression, as well as a partial rescue of the colony-forming ability (Fig. 2H, I, S10D) implying a role for MYC in PTEN repression also in H1299 cells. In LUAD patients, upregulation of EZH2, MYC and YY1 was associated with downregulation of PTEN expression, both at the protein and the RNA level. This association was specifically observed in patients harboring somatic alterations in the Hippo pathway and MYC-related pathways (Fig. S3A, B). Moreover, the expression level of PTEN protein negatively correlates with its putative transcriptional repressors TAZ, EZH2 and MYC while it positively correlates with pYAPS127 (the YAP phosphorylated form that is retained in the cytoplasm), as well as with the onco-suppressive transcripts TGFBR2, CDKN1A (p21) and CDKN2B (p15), which were previously shown to be repressed by YAP/TAZ and EZH2 (Fig. S3C) [18]. Finally, Kaplan Mayer survival analysis shows a better prognosis for LUAD patients expressing high levels of PTEN, especially in the earlier stages of the disease, supporting its role as an early prognostic onco-suppressor (Fig. S3D).

### EZH2 and MYC inhibition activates YAP and TAZ targets

The oncogenic role of EZH2 and MYC in the transcriptional repression of onco-suppressors suggests that their inhibition would reactivate those onco-suppressors. Several specific inhibitors of EZH2, which catalyzes tri-methylation of histone H3 at Lys 27 (H3K27me3), have been developed, and Tazemetostat has been approved by the FDA for epithelioid sarcoma [31], diffuse large B-cell lymphoma and relapsed or refractory follicular lymphoma [32]. While Tazemetostat is effective for the above cancer types, it is ineffective as monotherapy for other malignancies, especially for solid cancers, where cells show either innate or acquired resistance to this compound [33]. Huang et al. showed that Tazemetostat treatment of solid tumors induced a decrease in global H3K27me3, as expected, which was accompanied by an increase in total H3K27ac. It was, therefore, proposed that an oncogenic transcriptional reprogramming mediated by the interaction of the epigenetic protein MLL1 with the p300/CBP complex onto several oncogenes induces global H3K27ac upregulation upon H3K27me loss, driving the hyperactivation of genes belonging to oncogenic pathways that counteract the de-repression of onco-suppressor pathways [33].

**Fig. 3:**
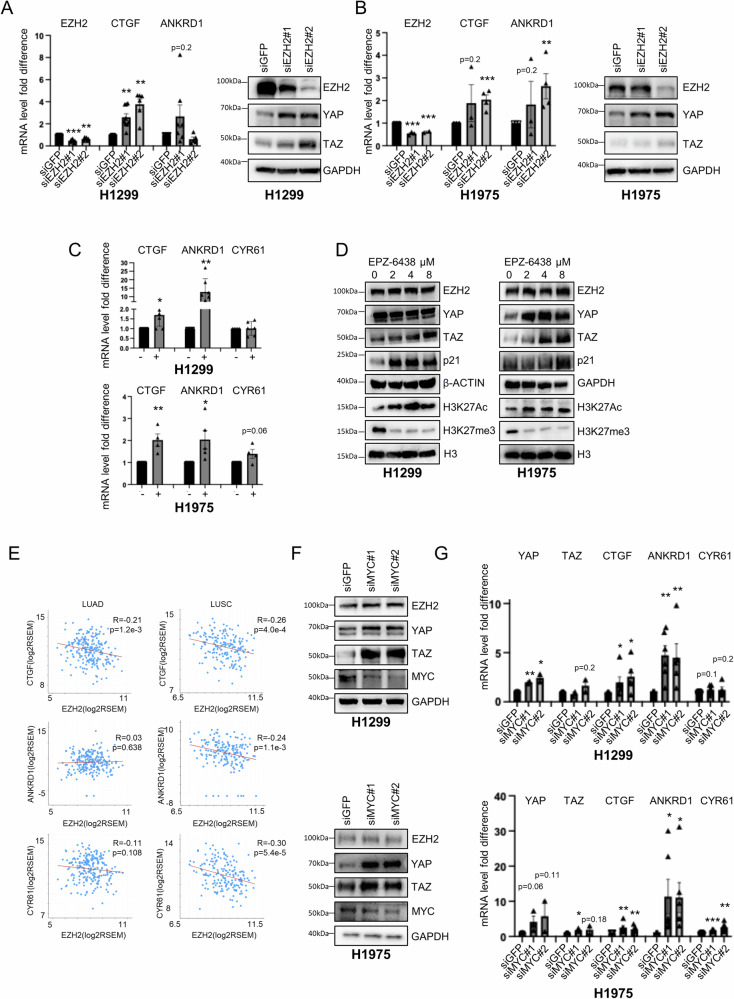
EZH2 and MYC inhibition activates YAP and TAZ targets. Real-time quantification (left) and WB analysis (right) of the indicated transcripts and proteins in H1299 (**A**) and H1975 (**B**) upon EZH2 KD. Charts represent the average and SEM of at least three independent biological replicates. Two-tailed *t*-test was applied to calculate the *p* values. *p < 0.05; **p < 0 .01; ***p < 0.001. H1299: EZH2 siEZH2#1 vs siGFP *p* = 3.9e − 5, *n* = 7. EZH2 siEZH2#2 vs siGFP *p* = 0.001, *n* = 6. CTGF siEZH2#1 vs siGFP *p* = 0.001, *n* = 9. CTGF siEZH2#2 vs siGFP *p* = 0.002, *n* = 6. ANKRD1 siEZH2#1 vs siGFP *p* = 0.2, *n* = 9. ANKRD1 siEZH2#2 vs siGFP *p* = 0.01, *n* = 5. H1975: EZH2 siEZH2#1 vs siGFP *p* = 0.0004, *n* = 3. EZH2 siEZH2# vs siGFP *p* = 0.0004, *n* = 3. CTGF siEZH2#1 vs siGFP *p* = 0.2, *n* = 4. CTGF siEZH2#2 vs siGFP *p* = 0.0002, *n* = 4. ANKRD1 siEZH2#1 vs siGFP *p* = 0.5, *n* = 4. ANKRD1 siEZH2#2 vs siGFP *p* = 0.002, *n* = 4. **C** Real-time quantification of CTGF, ANKRD1 and CYR61 transcripts upon treatment with 2 μM Tazemetostat (EPZ-64238) 6-day post-treatment in H1299 (top) and H1975 cells (bottom). Charts represent the mean and SEM of at least three independent biological replicates. Two-tailed *t*-test was used to calculate the *p* values. H1299 CTGF treated vs ctrl *p* = 0.02 *n* = 7. ANKRD1 treated vs ctrl *p* = 0.008, *n* = 7. CYR61 treated vs ctrl *p* = 0.9, *n* = 6. H1975 CTGF treated vs ctrl *p* = 0.004, *n* = 4. ANKRD1 treated vs ctrl *p* = 0.03, *n* = 5. CYR61 treated vs ctrl *p* = 0.06, *n* = 4. **D** WB analysis of the indicated proteins in H1299 (left) and H1975 cells (right) with or without treatment with the indicated doses of Tazemetostat for 6 days. **E** Dot plot showing the correlation between EZH2 transcript with the CTGF, ANKRD1 and CYR61 transcripts in lung adenocarcinoma patients (*n* = 517) and in lung squamous cell carcinoma (*n* = 484) as obtained from the cBioportal database by querying the lung adenocarcinoma TCGA, Firehose Legacy study or the Lung Squamous Cell Carcinoma TCGA, PanCancer Atlas, respectively. R indicates the Pearson’s correlation coefficient. WB (**F**) and real-time quantification (**G**) of the indicated proteins and transcripts in H1299 (top) and H1975 cell line (bottom) with or without MYC interference. For transcript analysis, mean and SEM are indicated. One-tailed *t*-test was applied to calculate the *p* value. *p* and *n* values in H1299: YAP siMYC#1 vs siGFP *p* = 0.007 *n* = 2. YAP siMYC#2 vs siGFP *p* = 0.03, *n* = 2. TAZ siMYC#1 vs siGFP *p* = 0.22, *n* = 2. TAZ siMYC#2 vs siGFP *p* = 0.2, *n* = 2. CTGF siMYC#1 vs siGFP *p* = 0.05, *n* = 6. CTGF siMYC#2 vs siGFP *p* = 0.02, *n* = 5. ANKRD1 siMYC#1 vs siGFP *p* = 0.001, *n* = 6. ANKRD1 siMYC#2 vs siGFP *p* = 0.01, *n* = 5. CYR61 siMYC#1 vs siGFP *p* = 0.11, *n* = 6. CYR61 siMYC#2 vs siGFP *p* = 0.2, *n* = 5. H1975: YAP siMYC#1 vs siGFP *p* = 0.06, *n* = 3. YAP siMYC#2 vs siGFP *p* = 0.11, *n* = 2. TAZ siMYC#1 vs siGFP *p* = 0.01, *n* = 3. TAZ siMYC#2 vs siGFP *p* = 0.18, *n* = 2. CTGF siMYC#1 vs siGFP *p* = 0.006, *n* = 6. CTGF siMYC#2 vs siGFP *p* = 0.007, *n* = 6. ANKRD1 siMYC#1 vs siGFP *p* = 0.02, *n* = 6. ANKRD1 siMYC#2 vs siGFP *p* = 0.01, *n* = 6. CYR61 siMYC#1 vs siGFP *p* = 9.1e-7, *n* = 6. CYR61 siMYC#2 vs siGFP *p* = 0.001, *n* = 6.

We previously showed that H1299 and H1975 cells are resistant to Tazemetostat, with an IC50 of 24 and 36 μM, respectively [18]. We also observed a reduced number of colonies in H1299 but not in H1975 or h358 cells at micromolar doses of Tazemetostat (Fig. S4A). In these cells, depletion of EZH2 by siRNA or treatment with 2 μM Tazemetostat increased the expression of YAP and TAZ and their transcriptional targets CTGF, ANKRD1 and CYR61 (Fig. 3A–C, S11A). These effects were also observed in H1299 and H1975 cells upon treatment with a lower dosage of Tazemetostat (1 μM, Figs. S4B, S13C, D). Moreover, Tazemetostat treatment induces a dose-dependent increase of global H3K27ac that counteracts the H3K27me3 decrease (Fig. 3D, S11B, C). This epigenetic switch was observed previously in other Tazemetostat-resistant cells but not in sensitive cell lines [33]. We found that global levels of YAP and TAZ are increased upon Tazemetostat treatment in a dose-dependent fashion, especially at high doses and more strongly in H1975 cells (Fig. 3D, S11B, C) which are more resistant to Tazemetostat than H1299 (Fig. S4A) [18]. In accordance, both in lung adenocarcinoma (LUAD) and in lung squamous cell carcinoma (LUSC) patients, the EZH2 mRNA has a negative correlation with CTGF, ANKRD1 and CYR61 transcripts (Fig. 3E).

Based on these results, we speculated that the upregulation of YAP- and TAZ-oncogenic signature may associate with the acquired resistance of H1299 and more strongly of H1975 cells to Tazemetostat treatment. Strikingly, the knockdown of YAP and TAZ increased the sensitivity of H1975 cells to very low doses of Tazemetostat (Fig. S4C) compared to Tazemetostat alone, which did not affect colony formation even at higher doses (up to 8 μM, Fig. S4A). Interestingly, MYC depletion or inhibition by MYCi975 also increased the expression of YAP and TAZ targets in H1299, H1975 and H358 cells (Fig. 3F, G, S4D, E, S11D, E, S13E, S14A) consistent with previous reports in liver and breast cancer models [24, 34]. Furthermore, the three cell lines were resistant to micromolar doses of MYCi975 as shown by the colony formation assay (Fig. S4F). In contrast, treatment with JQ1, a BRD4 inhibitor that indirectly inhibits oncogenic MYC function [35], induced opposite effects in different cell lines, increasing the expression of total YAP, TAZ, CTGF and ANKRD1 in H1299 but decreasing them in H1975 cells (Figs. S4G, S14B). This might be related to the broad effects of JQ1, which not only inhibits MYC but also other oncogenic pathways through epigenetic mechanisms. Notably, it was previously shown that combination of Tazemetostat with JQ1 inhibits MLL1-mediated H3K27ac accumulation onto oncogenes, and reverses the resistance to EZH2 inhibitors in different cellular models [33]. However, considering the different effects of JQ1 treatment in our models, we decided to inhibit MYC directly with MYCi975 and hypothesized that combined inhibition of YAP/TAZ together with MYC and EZH2 may be the best strategy to prevent activation of YAP/TAZ-mediated oncogenic pathways and concurrently de-repressing onco-suppressive pathways.

### Simultaneous inhibition of EZH2, MYC and YAP/TAZ maximizes the de-repression of onco-suppressors while preventing the activation of oncogenes

**Fig. 4:**
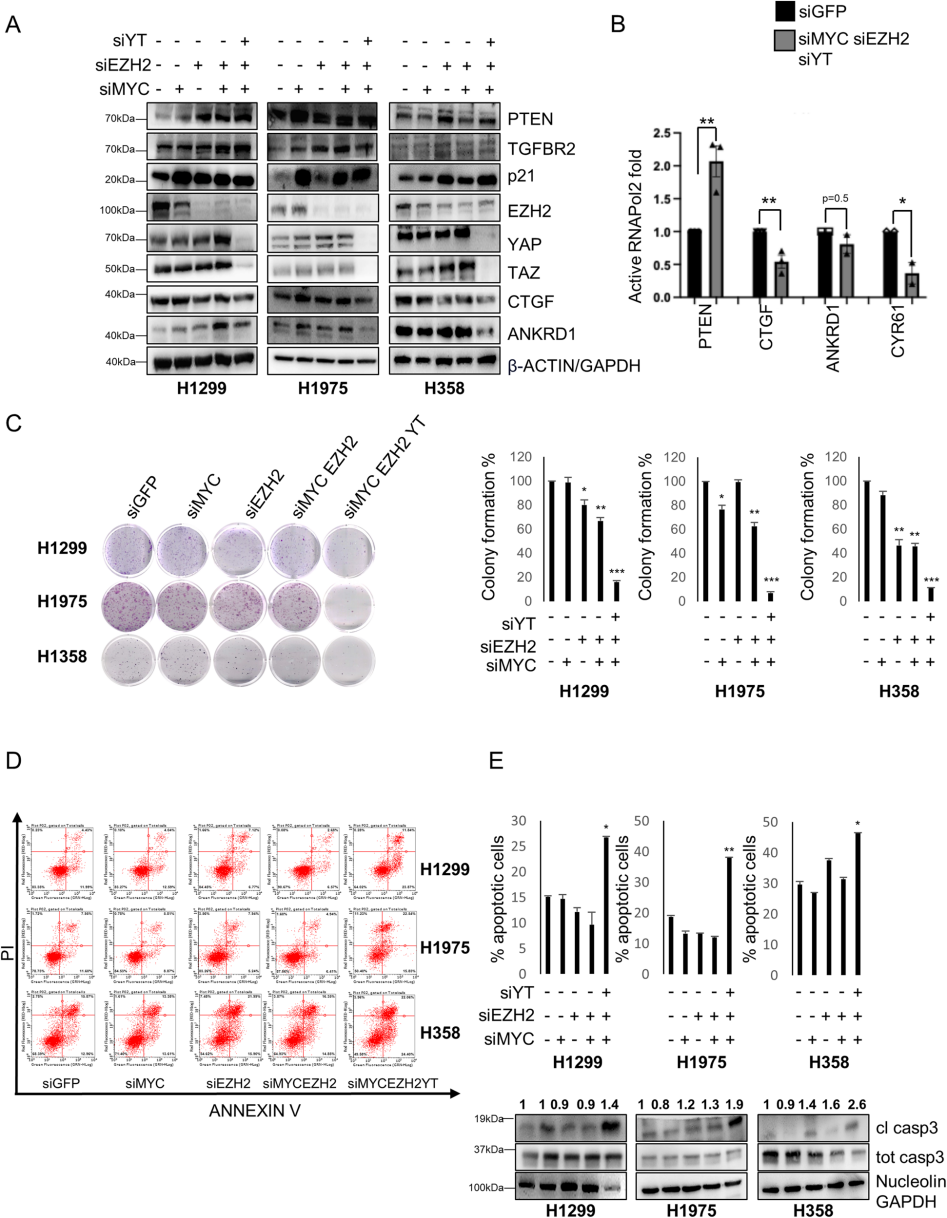
Simultaneous inhibition of EZH2, MYC and YAP/TAZ maximizes de-repression of onco-suppressors while contrasting activation of oncogenes. **A** WB of the indicated proteins in control and KD of MYC, EZH2 or YAP/TAZ, either alone or in combination, in H1299, H1975 and H358 cell lines. GAPDH or β-actin were used as loading control. **B** Immunoprecipitation analysis of the abundance of active RNAPol2 on the indicated promoters in H1299 cells concomitantly depleted for MYC, EZH2 and YAP/TAZ, compared to siGFP as control. Fold enrichment was calculated over IGG and then normalized to the siGFP control that was adjusted to 1. Mean, SEM and *p* value are represented. PTENp siMEY vs siGFP *p* = 0.005, *n* = 3. CTGFp siMEY vs siGFP *p* = 0.0004, *n* = 3. ANKRD1p siMEY vs siGFP *p* = 0.15, *n* = 2. CYR61p siMEY vs siGFP *p* = 0.03, *n* = 2. One-tailed *t*-test was applied to calculate the *p* value. **C** Representative images (left) and relative quantification (right) of colony formation in H1299, H1975 and H358 cells at the indicated interference conditions. Charts represent the mean and SEM of technical replicates from a representative biological replicate. The experiment was repeated three times. H1299 siEZH2 vs siGFP *p* = 0.04, *n* = 3. siMYC/siEZH2 vs siGFP *p* = 0.007, *n* = 3. siMYC/siEZH2/siYT vs siGFP *p* = 0.0001, *n* = 3. H1975 siMYC vs siGFP *p* = 0.02, *n* = 3. siMYC/siEZH2 vs siGFP *p* = 0.006, *n* = 3. siMYC/siEZH2/siYT vs siGFP *p* = 0.0001, *n* = 3. H358 siEZH2 vs siGFP *p* = 0.04, *n* = 3. siMYC/siEZH2 vs siGFP *p* = 0.007, *n* = 3. siMYC/siEZH2/siYT vs siGFP *p* = 0.0001, *n* = 3. **D** Scatter plot of the apoptotic rate, as measured through annexin V/PI staining, in H1299, H1975 and H358 cells at the indicated interference conditions. **E** Upper panel: quantification of the apoptotic rate, as measured through annexin V/PI staining, in H1299, H1975 and H358 cells at the indicated interference conditions. Charts represent the mean and SEM of technical replicates from a representative biological replicate. The experiment was repeated two or three times for each cell line. H1299 siMYC/siEZH2/siYT vs siGFP *p* = 0.01, *n* = 2. H1975 siMYC/siEZH2/siYT vs siGFP *p* = 0.006, *n* = 2. H1358 siMYC/siEZH2/siYT vs siGFP *p* = 0.03, *n* = 2. Lower panel: WB of the indicated proteins in control cells and KD of MYC, EZH2 or YAP/TAZ, either alone or in combination, in H1299, H1975 and H358 cell lines. Numbers above cleaved casp3 blot indicate the quantification of cleaved casp3 normalized to total casp3 signal. GAPDH or nucleolin were used as loading controls. *p < 0.05; **p < 0.01; ***p < 0.001.

We next depleted EZH2, MYC and YAP/TAZ by RNA interference (RNAi) either alone or in combination. We found that the triple knockdown (KD) of EZH2, MYC and YAP/TAZ led to stronger de-repression of onco-suppressor genes (TGFBR2, PTEN, p21) while reducing the activation of YAP and TAZ targets, which was observed upon double EZH2/MYC KD (Fig. 4A, S12A). Consistent with these results, we found that triple KD increased the binding of active RNAPol2 onto the PTEN promoter but decreased the binding onto the CTGF and CYR61 promoters (Fig. 4B). Functionally, the triple KD had stronger anti-tumorigenic effects compared to single or double KD in NSCLC cell lines, as shown by the reduced colony formation potential (Fig. 4C) and by the apoptotic phenotype demonstrated by Annexin V/PI staining and by cleaved casp3 (Fig. 4A, D, E, S5A, S12B). Conversely, KD of PTEN reduced the percentage of apoptotic cells already 24 h post-transfection (Fig. S5B) and the level of cleaved PARP/casp3 (Figs. S5C, S15). These results suggest that the increased apoptosis observed in response to triple KD is possibly mediated, at least in part, by the increase in PTEN and the concurrent decrease of YAP and TAZ. Moreover, PTEN KD increased colony formation in H1299, H1975 and H358 cells (Fig. S5D). To further confirm the KD results, we treated H1299 and H1975 cells with low doses of MYCi975 [36], Tazemetostat and Dasatinib (an indirect YAP and TAZ inhibitor) [37]. Low doses of the combined three inhibitors reduced the viability of both cell lines and de-repressed onco-suppressor genes while suppressing reactivation of YAP and TAZ oncogenes (Figs. S6A, B, S16) consistent with the KD experiments.

### Knockdown of PTEN restores the tumorigenic properties of MYC, EZH2 and YAP/TAZ depleted NSCLC cells

**Fig. 5:**
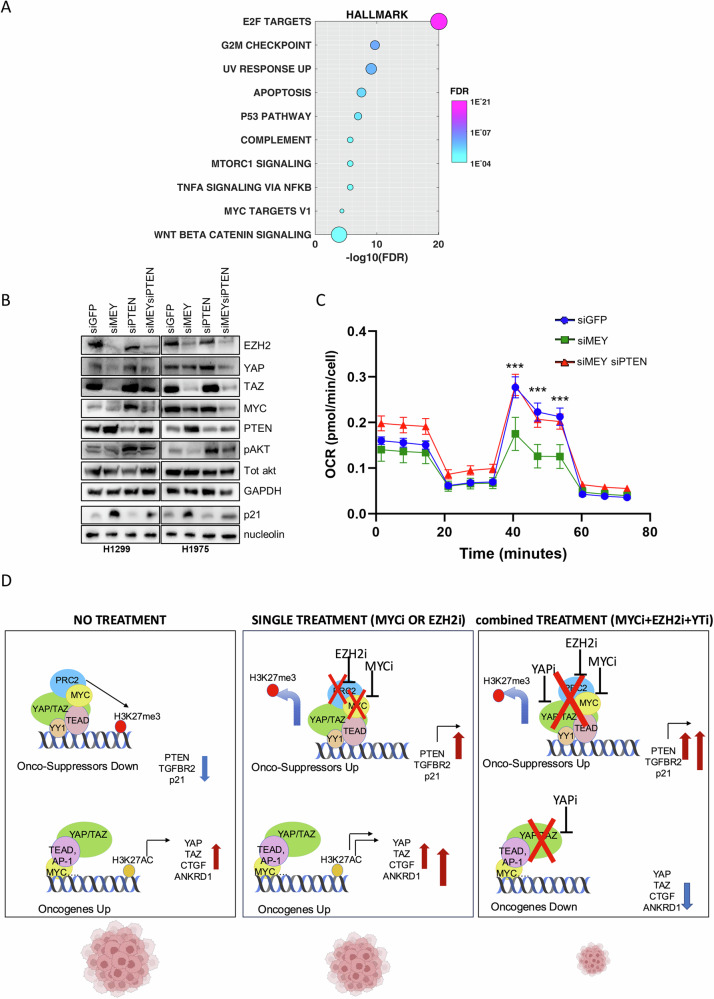
PTEN interference reverses phenotypes induced by MYC, EZH2 and YAP/TAZ depletion. **A** Gene set enrichment analysis (GSEA) of proteins significantly anti-correlated to PTEN in LUAD as obtained from cBioportal and listed in Table S2. The data set used for the analysis is the Lung Adenocarcinoma Firehose Legacy. FDR for each pathway is indicated. **B** WB of the indicated proteins at the indicated interference conditions in H1299 and H1975 cells. **C** Mitochondrial respiration measured as oxygen consumption rate through Sea Horse HS Mini Analyzer at the indicated interference conditions in H1299 cells. Data are represented as mean and SEM of three independent biological replicates. One-way ANOVA was applied to calculate the *p* value. siGFP vs siMEY *p* < 0.0001, *n* = 3 siMEY vs siMEY siPTEN *p* = 0.0003, *n* = 3. (3 is the number of the biological replicates, each of them has technical replicates). **D** Working model. In cancer, YAP and TAZ elicit their oncogenic role both through the inhibition of onco-suppressor genes and the activation of oncogenes, depending on associated transcriptional partners and epigenetic complexes. MYC and EZH2 cooperate with YAP and TAZ in the repression of onco-suppressor genes. The Inhibition of MYC and EZH2 de-represses onco-suppressor genes. However, YAP and TAZ activity increases onto oncogenic targets, which become hyper-activated. This can explain the resistance of solid cancers to the employment of MYC or EZH2 inhibitors as single agents. Concomitant inhibition of MYC, EZH2 and YAP/TAZ, which causes both the de-repression of onco-suppressors and the inhibition of oncogenes, is a more effective treatment in lung cancer models.

To further demonstrate the link between the three transcriptional regulators: MYC, EZH2 and YAP/TAZ and the tumor suppressor PTEN, we downloaded a list of transcripts and proteins positively or negatively correlated to PTEN in LUAD patients from the lung adenocarcinoma TCGA, Firehose Legacy study (Table S2). Gene set enrichment analysis (GSEA) of anti-correlated proteins highlighted E2F targets, mTORC1 signaling, and MYC targets as significantly anti-correlated to PTEN, consistent with the roles of PTEN in cell proliferation, cell growth, and cell metabolism (Table S2 and Fig. 5A).

To demonstrate that inhibition of PTEN expression through the concerted function of MYC, EZH2, and YAP/TAZ contributes to NSCLC tumorigenicity, we knocked down PTEN together with MYC, EZH2, and YAP/TAZ (defined as “MEY”). MEY KD induced PTEN and p21 reactivation and concurrently inhibited the PI3K/AKT/mTOR pathway as shown by reduced pAKT levels (Fig. 5B, S13A). These effects were accompanied by reduced mitochondrial respiration (Fig. 5C), which was previously shown to be essential for early events of lung tumorigenesis [38]. Strikingly, the simultaneous KD of PTEN could partially rescue these cellular changes (Fig. 5B, C, S7, S13A).

Altogether, these observations are summarized in Fig. 5D: in cancer context, onco-suppressors are repressed while oncogenes are hyper-activated due to the aberrant functions of EZH2, MYC and YAP/TAZ. Treatment with Tazemetostat (or with MYC inhibitors) as a single agent, reactivates onco-suppressors on one hand but concomitantly may induce stronger activation of oncogenic signaling, including the activation of YAP/TAZ signature, as a mechanism of acquired resistance. Targeting of YAP/TAZ oncogenic signature may overcome the acquired resistance to Tazemetostat and/or MYC inhibitors. Combined targeting of EZH2, MYC and YAP/TAZ can, therefore, effectively de-repress expression of tumor suppressors and concurrently repress the expression of oncogenic pathways, to ultimately inhibit tumor growth.

To explore the possibility that this mechanism is conserved across other tumor types, we analyzed PTEN expression in various tumors using the Firebrowse Gene Expression Viewer, which provides access to genomic data from The Cancer Genome Atlas (TCGA). From this analysis, it is evident that PTEN expression is downregulated in several tumor types with a sufficient number of patients, except in stomach cancer, HNSCC (Head and Neck Squamous Cell Carcinoma), and renal cancers (Fig. S8A). In tumors where PTEN is downregulated, the fold change in expression between tumoral and normal tissue ranges from 0.5 in GBM (Glioblastoma Multiforme) to 0.89 in THCA (Thyroid Carcinoma). The analysis of 273 Glioblastoma Multiforme patients from the TCGA Firehose Legacy cohort, for which mutation and CNA (Copy Number Alteration) data are available, reveals that PTEN is genetically altered with a frequency of 41% (Fig. S8B). Moreover, PTEN is downregulated at both the transcript and protein levels in tumoral tissue compared to its non-tumoral counterpart (Fig. S8C). Conversely, YAP, MYC and EZH2 are upregulated in GBM tumoral tissue and show an inverse correlation with PTEN in patients (Fig. S8C, D). Analyzing a cohort of GBM/LGG patients from the TCGA, we observed that in GBM patients, who are advanced grade (grade IV), high expression of PTEN correlates with a better prognosis, although the correlation is a non-significant trend. However, in LGG (Low Grade Glioma), the prognostic power of PTEN is stronger and statistically significant (Fig. S8E). This is consistent with our observations in LUAD patients, where PTEN shows greater prognostic value in the earlier stages of the disease (Fig. S3D).

In line with these observations, recent studies have provided accumulating evidence of an onco-suppressive role for PTEN in GBM, through the inhibition of proliferation and metastasis, as well as the promotion of genomic stability and immune response [39]. Moreover, EZH2 was found to inhibit PTEN expression in GBM by binding to its promoter and inducing H3K27me3 modification [40]. This expands our findings in another cancer model, where the more pronounced downregulation of PTEN may result from a combination of genetic alterations and transcriptional repression mediated by the YAP/MYC/EZH2 repressive complex.

## Discussion

In this study, we highlight the role of YAP/TAZ not only as transcriptional co-activators of oncogenes but also as transcriptional co-repressors of onco-suppressor genes. Previous studies demonstrated that YAP can function as a transcriptional repressor in breast [41], Schwann cells, trophoblast and intestine, through different mechanisms [17–20]. In breast, YAP was found at the promoter of a subset of genes, including those of the 1q21.3 locus. In that context, a complex of YAP, TEAD4, AIB1 and ANCO1 functions as a transcriptional repressor in normal breast cells, while during tumorigenesis, the loss of ANCO1 leads to a switch from transcriptional repression toward transcriptional activation [41]. Furthermore, the Nucleosome Remodeling Deacetylase (NuRD) complex mediates the transcriptional repressive function of YAP and TAZ on several target genes, including DDIT4, a negative regulator of mTORC1 [42]. Our work provided a novel mechanism mediated by YAP and TAZ to sustain the mTOR pathway, specifically through PTEN downregulation directly at the transcriptional level, in addition to previous works highlighting DDIT4 repression [42] or the upregulation of miR29 that leads to post-transcriptional repression of PTEN [43]. Our newly characterized repression mechanism introduces new players, the EZH2 methyltransferase and the MYC transcription factor. EZH2 was found to co-repress onco-suppressor genes with YAP also in Schwann cells [19] and in breast cancer cells [44] as well as inflammation-related genes in intestinal cells [20]. In the study by Wang et al., YAP was shown to recruit EZH2 onto GDF15 promoter, resulting in its repression and metastatization of breast cancer cells [44], suggesting that this mechanism of transcriptional repression might be conserved in different cell types. The discovery of the EZH2/MYC/YAP/TAZ oncogenic axis provides the rationale for the inhibition of these proteins, and we found in vitro that the best functional effect can be obtained through the simultaneous inhibition of all these players.

## Materials and methods

### Cell lines used for in vitro experiments

NCI-H1299 (RRID:CVCL0060), NCI-H1975 (RRID:CVCL_1511), NCI-H358 (RRID:CVCL_1559) were purchased from the American Type Culture Collection (ATCC, Manassas, VA), authenticated by digital PCR, and routinely tested by PCR for mycoplasma contamination by using the following primers: Myco_fw1: 5′-ACACCATGGGAGCTGGTAAT-3′, Myco_rev1: 5′-CTTCATCGACTTTCAGACCCAAGGCA-3′. Cells were grown in RPMI medium (Invitrogen, Carlsbad, CA) supplemented with 10% fetal bovine serum and Pen/Strep antibiotic at 37 °C in a balanced air-humidified incubator with 5% CO_2_. Lipofectamine RNAimax (Invitrogen) was used in accordance with the manufacturer’s instruction for transfection with siRNAs. SiRNAs were used at the final amount of 300 pmol in 60 mm dish. List of siRNAs used for functional in vitro experiments is given below. Cells were collected 48–72 h post-transfection for subsequent analyses. All cell lines have been authenticated in the past 3 years.

### Transient transfection with plasmids

The plasmid for MYC overexpression was obtained by cloning the human MYC cDNA into pCDNA3 backbone and was a kind gift from the laboratory of Dr. Maurizio Fanciulli (IRCCS Regina Elena, Rome, Italy). pCDNA empty vector was used as control. Cells in suspension were transfected with siGFP or siYAP/TAZ using Lipofectamine RNAimax. 24 h post-transfection, cells in adhesion were transiently transfected with pCDNA3 empty vector of with pCDNA3-MYC using Lipofectamine 2000 according to manufacturer instructions. Cells were collected 48 h after plasmid transfection for subsequent analyses. Plasmid for PTEN overexpression was obtained by cloning the PTEN wt cDNA into pEGFP-C2 backbone and was a kind gift of Dr. Fabiana Conciatori (IRCCS Regina Elena, Rome, Italy). Empty vector was used as negative control.

### Clonogenic assay

Cells were transfected as indicated above and 48–72 h later they were detached and seeded at 500–1000 cells/well into 6-well or 12-well dishes. Fresh medium was added every 4 days. After 7–14 days, colonies were stained with crystal violet and counted.

For colony assay upon treatment, cells were seeded in triplicate at a density of 1000 cells/well in a 6-well multiwell with or without Tazemetostat or MYCi975 at the indicated doses. Every 3–4 days, fresh medium was added with or without Tazemetostat or MYCi975, then after 10–14 days the colonies were stained with crystal violet and counted. Two-tailed *t*-test analysis was applied to calculate the *P* values.

### Analysis of cell-cycle profile

For cell-cycle analysis, cells were collected 48–72 h after PTEN overexpression and fixed in 70% ethanol overnight. Fixed cells were washed and treated with RNase at 1 mg/ml final concentration for 30 min at 37 °C before adding 5 mg/ml PI and analyzed with the Attune flow cytometer (Invitrogen) and FlowJo software.

### Annexin V/PI assay

Analysis of apoptotic cells upon different interference and treatment conditions was performed by using the Annexin V-FITC Apoptosis Kit 300 (Invitrogen #BMS500FI-300) following manufacturer instructions. Stained cells were analyzed with a Guava Easycyte 8HT flow cytometer equipped with Guava Soft 2.1 (Millipore) according to the manufacturer’s instructions.

### Pharmacological treatment and chemical reagents

Dasatinib (#S1021), Tazemetostat (EPZ-6438 #S7128), JQ1 (#S7110) and MYCi975 (#S8906) were obtained from Selleck Chemicals (Houston, TX).

### Protein extracts and western blot analysis

For the preparation of whole-cell lysates, cells were lysed in buffer with 50 mM Tris-HCl pH 7.6, 0.15 M NaCl, 5 mM EDTA, 1% Triton X-100 and fresh protease inhibitors. Extracts were sonicated for 10 + 15 s at 80% amplitude and centrifuged at 12,000~ rpm for 10 min to remove cell debris. Protein concentrations were determined by colorimetric assay (Bio-Rad). Antibodies used for western blotting are listed in the Supplementary Materials. Primary antibodies were diluted 1:1000, while secondary antibodies were diluted 1:10,000 in Tris-Buffered Saline (TBS) with 5% BSA and 0.1% Tween® 20 detergent.

### Transcript analysis

Total RNA was extracted using TRIzol (Ambion) according to the manufacturer’s recommendations. For gene transcript analysis, 1γ RNA was retrotranscribed using M-MLV reverse transcriptase (Invitrogen) following the manufacturer's instructions. Real-time PCR was performed into a final volume of 10 μl using Sybr Green PCR master mix, and normalized on GAPDH. All the real-time PCR assays were performed by using an Applied Biosystems® 7500 fast or a StepOne Real-Time PCR Instrument. Each analysis was performed at least on three independent biological replicates and two-tailed *t*-test analysis was applied to calculate the *P* values. List of primers used for real-time PCR is given in the Supplementary Materials.

### Chromatin immunoprecipitation (ChIp)

ChIP-qPCR was performed as described previously [45]. Briefly, cells were fixed with 1% formaldehyde (Sigma) in culture medium for 10 min at room temperature, and chromatin from lysed nuclei was sheared to 200–600 bp fragments using a Bioruptor Sonicator (Diagenode). 100 μg of sheared chromatin and 5 μg of antibody plus 40 μl of magnetic beads (Dynabeads® Protein G 10004D Thermo Fisher) were used for each Ip. Quantitative real-time PCR was carried out with Applied Biosystems® 7500 fast or StepOne Real-Time PCR Instruments. Each sample was analyzed in triplicate. The amount of immunoprecipitated DNA in each sample was determined as the fraction of the input (or mock) [amplification efficiency (Ct INPUT–Ct ChIP)], and normalized to the IgG control.

### Co-immunoprecipitation (Co-IP)

Co-IP was performed as described previously [46].

### Mitochondrial respiration analysis

For mitochondrial respiration analysis, cells were collected 48 h after interference and seeded in a Seahorse XFp PDL Miniplate (Agilent) to grow overnight in fresh medium. The next day, after a 30-min calibration of the XF sensor with the preincubated sensor cartridge, the cell plate was loaded into the analyzer, and oxygen consumption rate (OCR) was analyzed under basal conditions. Net mitochondrial oxygen consumption was then analyzed by sequential injection of the complex inhibitors oligomycin (1 µM), FCCP (1.2 µM), and the mixture of rotenone and antimycin A (0.5 μM each). Cells were stained with DAPI and the cell count of each well was determined by imaging the cells using Cell Profiler. Three replicates of each sample were analyzed two-tailed *t*-test analysis was applied to calculate the *P* values.

### Immunofluorescence

Cells were seeded overnight onto cover glasses in multiwell at different confluence. The next day cells were washed twice in Tris-Buffered Saline (TBS), fixed in 4% paraformaldehyde (PFA) in TBS for 10 min at RT and washed three times in TBS for 5 min at RT. Then cells were permeabilized with 0.1% Triton X-100 in TBS for 10 min at RT, washed three times in TBS for 5 min at RT, blocked in 3% BSA in TBS-T (0.1% Tween) for 45′ at RT. After blocking, cells were incubated with primary antibody overnight in humidity chamber at 4 °C. Primary antibody dilution ranges from 1:25 to 1:500 depending on the antibody. The next day, cells were washed three times in TBS-T for 5′ at RT and incubated with secondary antibody (1:500 dilution) for 1 h at RT in the dark, then washed three times in TBS-T 5′ at RT in the dark and incubated with Hoechst 1:2000 in TBS for 10′ at RT in the dark. Finally, cover glasses with cells were washed three times in TBS-T for 5′ at RT in the dark, mounted with 10 μl of 70% glycerol onto microscope slides and analyzed when dried. PLA assay was performed using a Duolink in situ PLA kit (Sigma-Aldrich) and following manufacturing instructions.

### Confocal microscopy and image analysis

Fixed and stained cells were imaged using a Zeiss 710 laser-scanning microscope under 63× Plan Apochromat (1.4 numeric aperture) oil immersion lens with Zen 2008 imaging software (Zeiss). Stacks were collected in 0.5-μm Z-section increments, and a representative stack is shown for each image.

### Opera Phenix Plus platform

For immunofluorescence experiments in H1975 cells, 500–100 cells/well were plated in 96-well phenoplates (Revvity). Images were acquired using the Opera Phenix Plus (Revvity) platform with a 20× water objective. The following excitation laser were used: 375 nm for the identification of nuclei stained with Hoechst 33342 (Sigma), 488 nm for the identification of YAP (rabbit), pMYC, YY1 and H3K27me3, incubated with anti-rabbit secondary antibody conjugated with Alexa Fluor 488 (Invitrogen), 640 nm for the identification of YAP (mouse) and Lamin A/C incubated with anti-mouse secondary antibody conjugated with Alexa Fluor 647 (Invitrogen). The Intensity of fluorescence and their co-localization were analyzed using Harmony software (Revvity).

### Bioinformatic analysis

Targets of MYC in A549 cell line were identified through the online database ChiP-Atlas (https://chip-atlas.org/). A hierarchical clustering analysis was performed using the targets modulated after silencing of YAP and TAZ. The RNAseq data used for this analysis was obtained from the Gene Expression Omnibus (GEO) with the accession number GSE151200. Genes obtained from GSE151200 were matched with MYC targets, (ChiP-Atlas), and also with genes bound by either EZH2 [28] or H3K27me3 (GSE29611) in A549 NSCLC cells. To gain insights into the biological pathways associated an enrichment pathway analysis was conducted. ShinyGO 0.77, a web-based tool (http://bioinformatics.sdstate.edu/go/), was utilized for this purpose.

### Curves of OS

Curves of overall survival in lung cancer patients were evaluated by Kaplan–Meier method by the Kaplan Mayer plotter tool (kmplot.com). Curves of patients with high and low signals were established by median value. Statistical significance between curves was assessed by using the logrank test.

### Analysis of differentially expressed genes

Transcripts or proteins differentially expressed in tumoral vs non-tumoral tissue in NSCLC patients were obtained from the UALCAN database (https://ualcan.path.uab.edu/analysis.html). Pathway alteration was measured through systematic evaluation of pathway-level somatic alterations by small mutation or copy number alteration across tumors with combined proteomic, whole-exome, and CNA data, involving key pathways and genes previously annotated across multiple cancer types based on domain knowledge (pathway annotations from PMID: 33568653).

### Correlation analyses

Correlation analyses between proteins and transcripts were performed through the cBioportal Database (https://www.cbioportal.org).

## Supplementary information

SUPPLEMENTARY FIGURES

TABLE S1

TABLE S2

SUPPLEMENTARY MATERIAL

## Data Availability

The gene expression and Chip-Seq data analyzed during the current study are available in the Gene Expression Omnibus (GEO) repository under accession number GSE151200 (genes upregulated upon YAP or TAZ interference in A549 cells), GSE29611 (genes enriched of H3K27me3 in A549 cells) and Chip-Atlas (MYC targets in A549 cells: https://chip-atlas.org/).
