# Supplementary material for: The oncogenic axis YAP/MYC/EZH2 impairs PTEN tumor suppression activity enhancing lung tumorigenicity
Source: Cell Death Discov. 2024 Oct 25;10:452. doi: 10.1038/s41420-024-02216-8 (PMC11511861; doi:10.1038/s41420-024-02216-8)

Fig. S1

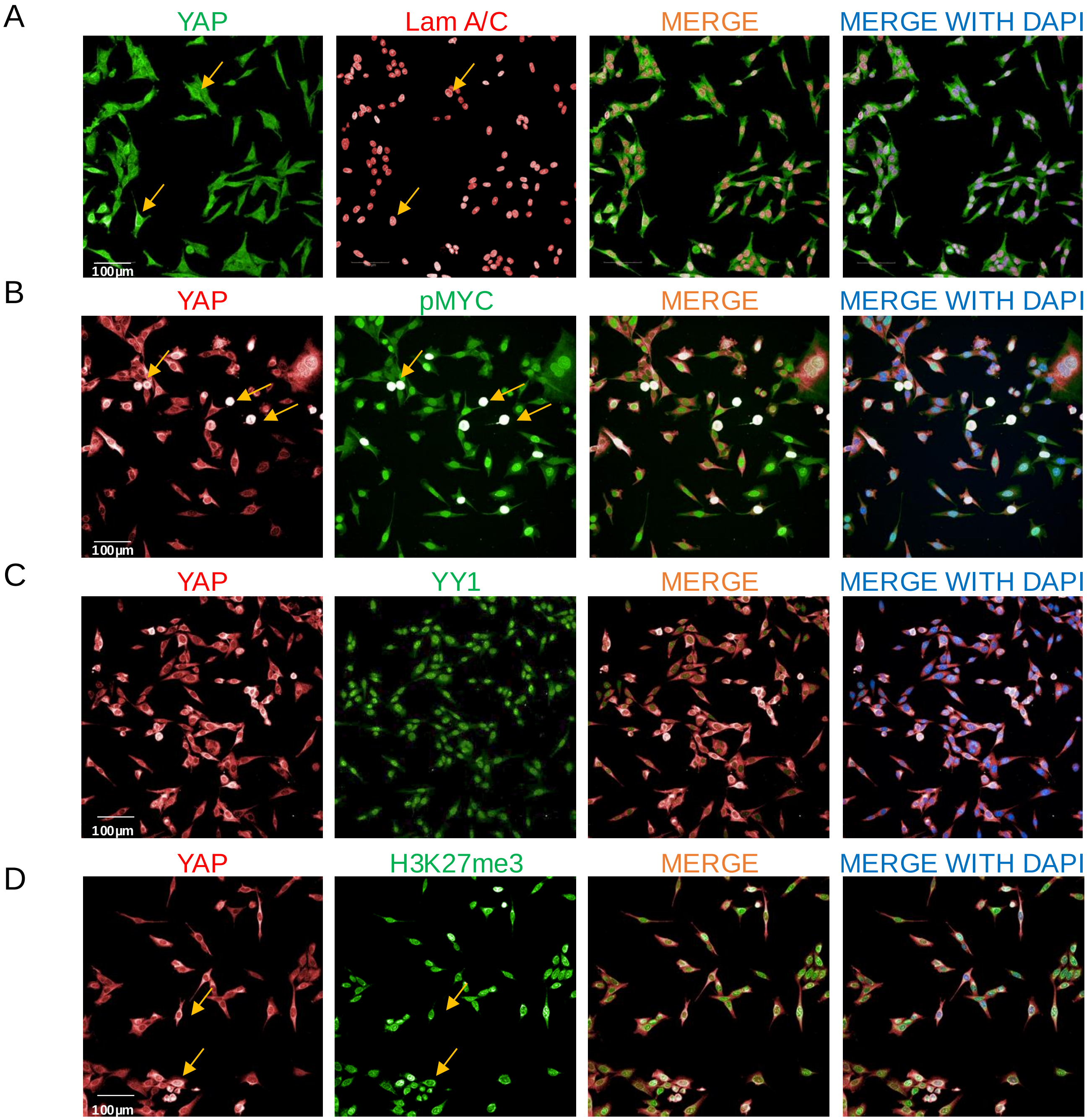
A
YAP
Lam A/C
MERGE
MERGE WITH DAPI
100µm
B
YAP
pMYC
MERGE
MERGE WITH DAPI
100µm
C
YAP
YY1
MERGE
MERGE WITH DAPI
100µm
D
YAP
H3K27me3
MERGE
MERGE WITH DAPI
100µm

Fig. S2

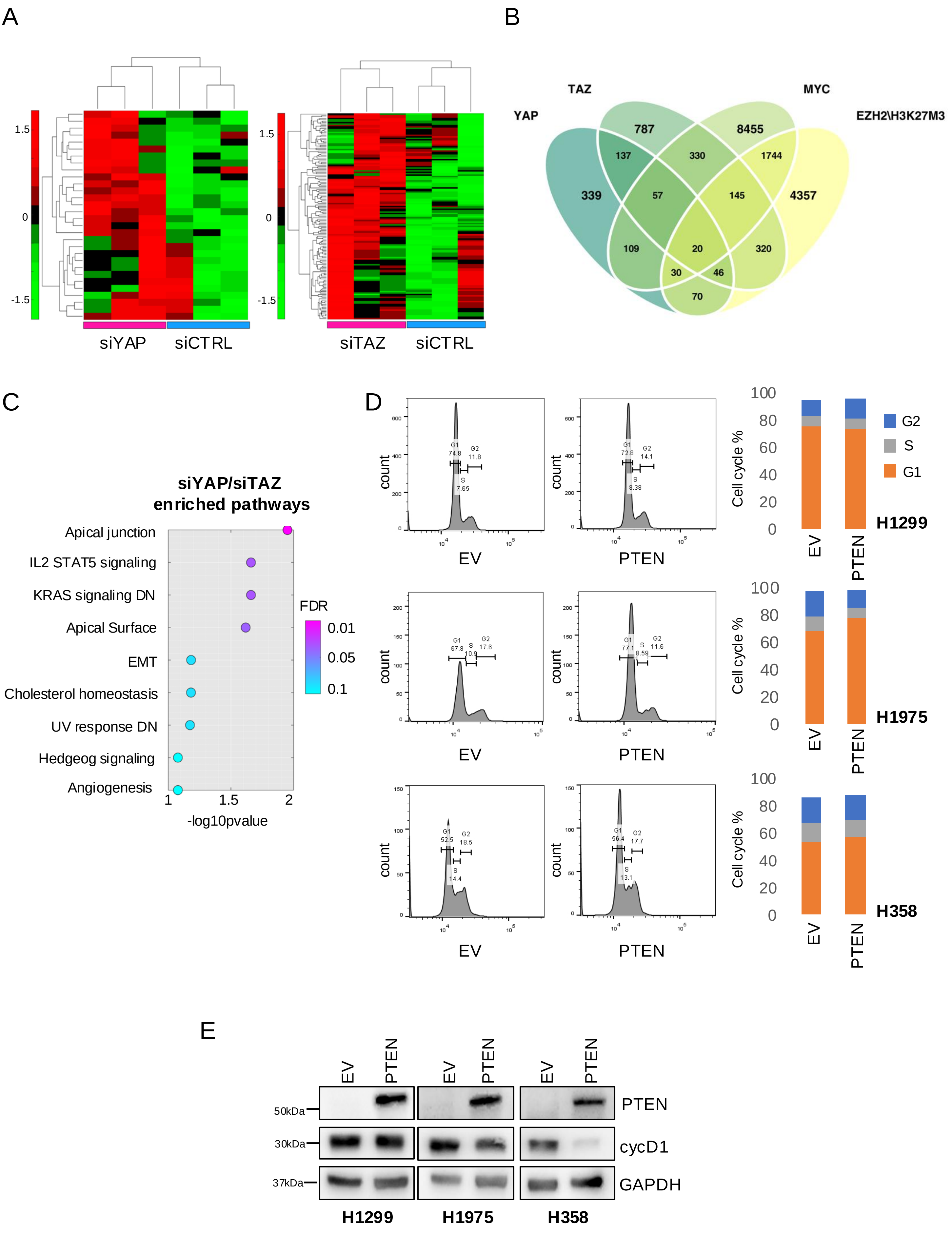
A
1.5
0
-1.5
siYAP
siCTRL
siTAZ
siCTRL
B
YAP
TAZ
MYC
EZH2\H3K27M3
339
787
8455
4357
137
330
1744
57
145
109
20
320
30
46
70
C
siYAP/siTAZ
enriched pathways
Apical junction
IL2 STAT5 signaling
KRAS signaling DN
Apical Surface
EMT
Cholesterol homeostasis
UV response DN
Hedgeog signaling
Angiogenesis
FDR
0.01
0.05
0.1
1
1.5
2
-log10pvalue
D
count
EV
PTEN
Cell cycle %
G2
S
G1
H1299
H1975
H358
E
EV
PTEN
50kDa
30kDa
37kDa
PTEN
cycD1
GAPDH
H1299
H1975
H358

Fig. S3

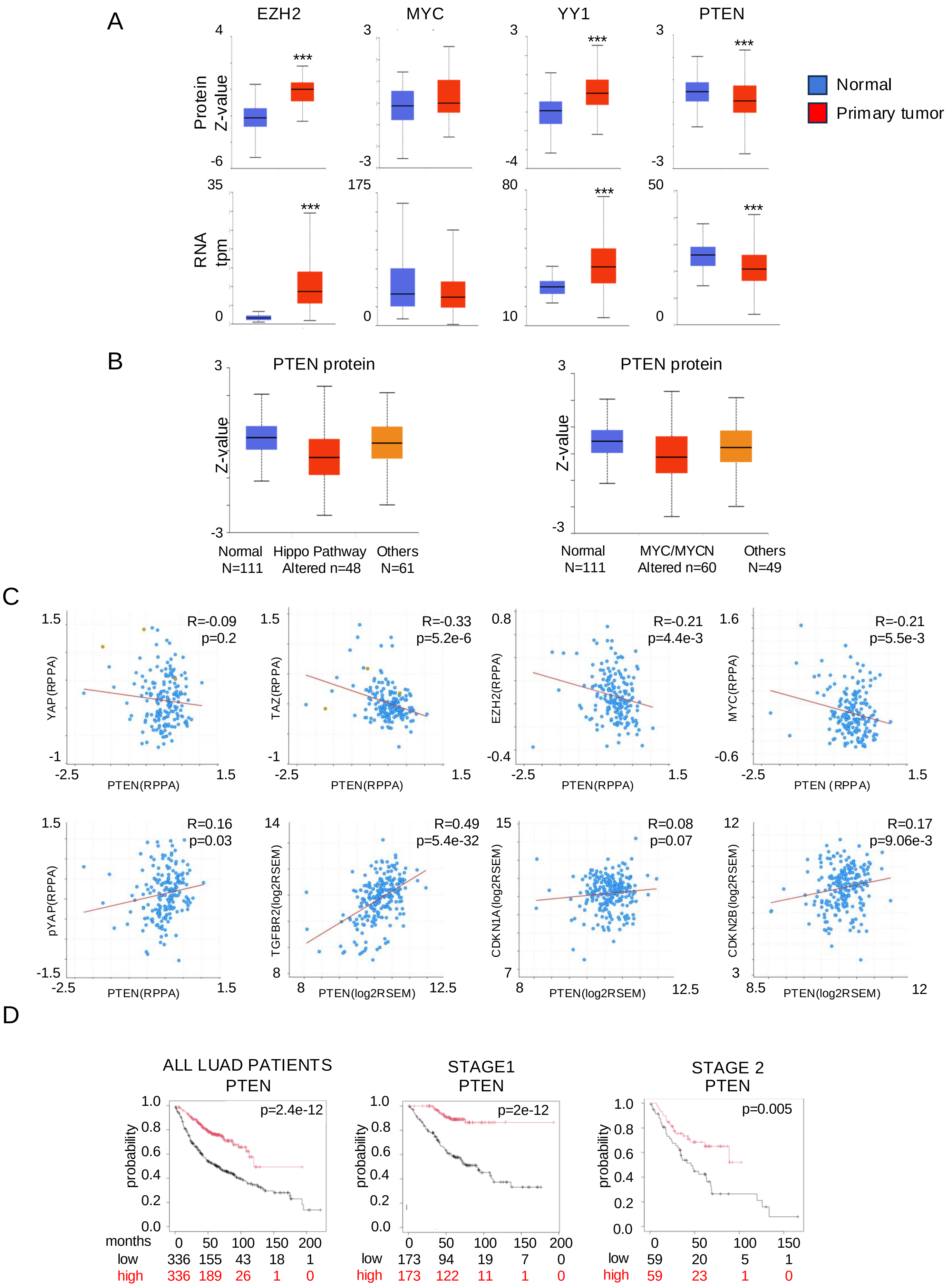
A
EZH2
MYC
YY1
PTEN
Protein
Z-value
4
-6
3
-3
3
-4
3
-3
***
***
***
Normal
Primary tumor
RNA
tpm
35
0
175
0
80
10
50
0
***
***
***
B
PTEN protein
3
-3
Z-value
Normal
N=111
Hippo Pathway
Altered n=48
Others
N=61
PTEN protein
3
-3
Z-value
Normal
N=111
MYC/MYCN
Altered n=60
Others
N=49
C
YAP(RPPA)
1.5
-1
-2.5
1.5
PTEN(RPPA)
R=-0.09
p=0.2
TAZ(RPPA)
1.5
-1
-2.5
1.5
PTEN(RPPA)
R=-0.33
p=5.2e-6
EZH2(RPPA)
0.8
-0.4
-2.5
1.5
PTEN(RPPA)
R=-0.21
p=4.4e-3
MYC(RPPA)
1.6
-0.6
-2.5
1.5
PTEN (RPPA)
R=-0.21
p=5.5e-3
pYAP(RPPA)
1.5
-1.5
-2.5
1.5
PTEN(RPPA)
R=0.16
p=0.03
TGFBR2(log2RSEM)
14
8
8
12.5
PTEN(log2RSEM)
R=0.49
p=5.4e-32
CDKN1A(log2RSEM)
15
7
8
12.5
PTEN(log2RSEM)
R=0.08
p=0.07
CDKN2B(log2RSEM)
12
3
8.5
12
PTEN(log2RSEM)
R=0.17
p=9.06e-3
D
ALL LUAD PATIENTS
PTEN
p=2.4e-12
probability
1.0
0.8
0.6
0.4
0.2
0.0
months
0 50 100 150 200
low 336 155 43 18 1
high 336 189 26 1 0
STAGE1
PTEN
p=2e-12
probability
1.0
0.8
0.6
0.4
0.2
0.0
0 50 100 150 200
low 173 94 19 7 0
high 173 122 11 1 0
STAGE 2
PTEN
p=0.005
probability
1.0
0.8
0.6
0.4
0.2
0.0
0 50 100 150
low 59 20 5 1
high 59 23 1 0

Fig. S4

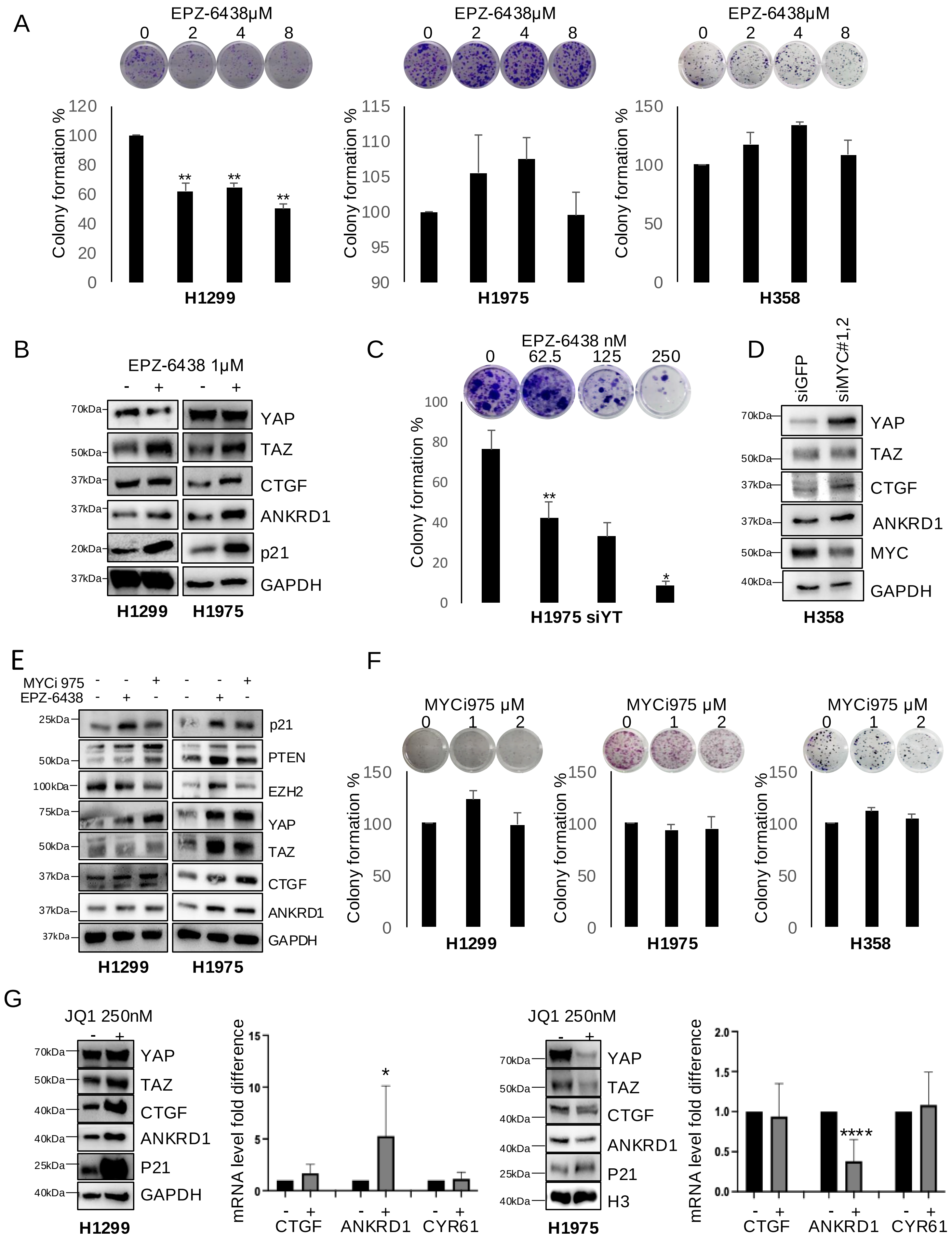

A
EPZ-6438μM
0 2 4 8
Colony formation %
H1299
H1975
H358
B
EPZ-6438 1μM
YAP
TAZ
CTGF
ANKRD1
p21
GAPDH
H1299 H1975
C
EPZ-6438 nM
0 62.5 125 250
H1975 siYT
D
siGFP
siMYC#1,2
YAP
TAZ
CTGF
ANKRD1
MYC
GAPDH
H358
E
MYCi 975
EPZ-6438
p21
PTEN
EZH2
YAP
TAZ
CTGF
ANKRD1
GAPDH
H1299 H1975
F
MYCi975 μM
0 1 2
H1299
H1975
H358
G
JQ1 250nM
YAP
TAZ
CTGF
ANKRD1
P21
GAPDH
H1299
mRNA level fold difference
CTGF ANKRD1 CYR61
H3
H1975

Fig. S5

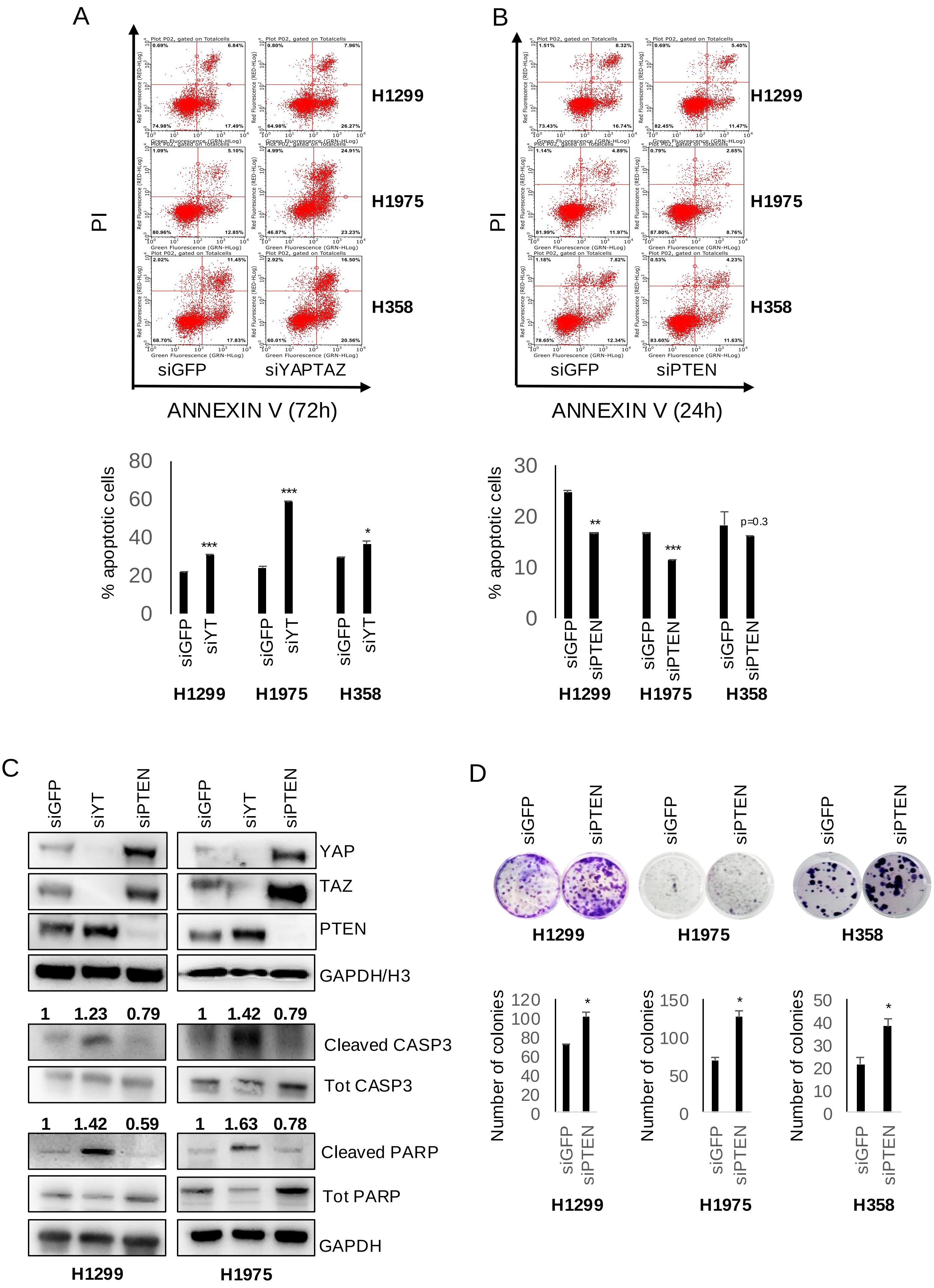

A
PI
H1299
H1975
H358
siGFP
siYAPTAZ
ANNEXIN V (72h)
B
PI
H1299
H1975
H358
siGFP
siPTEN
ANNEXIN V (24h)
% apoptotic cells
80
60
40
20
0
***
***
*
siGFP
siYT
H1299
H1975
H358
% apoptotic cells
30
20
10
0
**
***
p=0.3
siGFP
siPTEN
H1299
H1975
H358
C
siGFP
siYT
siPTEN
YAP
TAZ
PTEN
GAPDH/H3
1 1.23 0.79
1 1.42 0.79
Cleaved CASP3
Tot CASP3
1 1.42 0.59
1 1.63 0.78
Cleaved PARP
Tot PARP
GAPDH
H1299
H1975
D
siGFP
siPTEN
H1299
H1975
H358
Number of colonies
120
100
80
60
40
20
0
150
100
50
0
50
40
30
20
10
0
*
*
*
siGFP
siPTEN
H1299
H1975
H358

Fig. S6

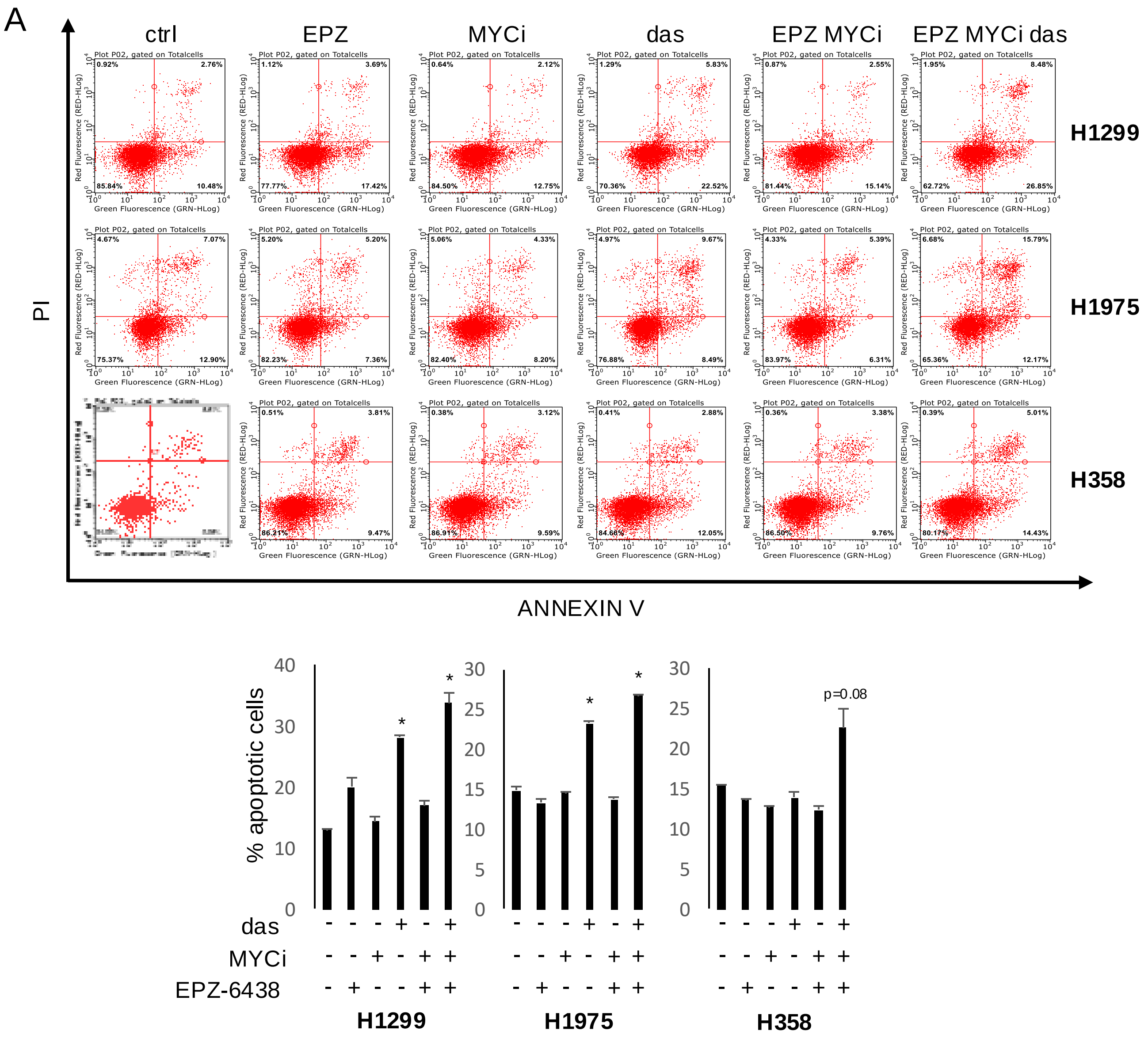

A
ctrl
EPZ
MYCi
das
EPZ MYCi
EPZ MYCi das
H1299
H1975
H358
PI
ANNEXIN V
% apoptotic cells
p=0.08
das
MYCi
EPZ-6438
H1299
H1975
H358

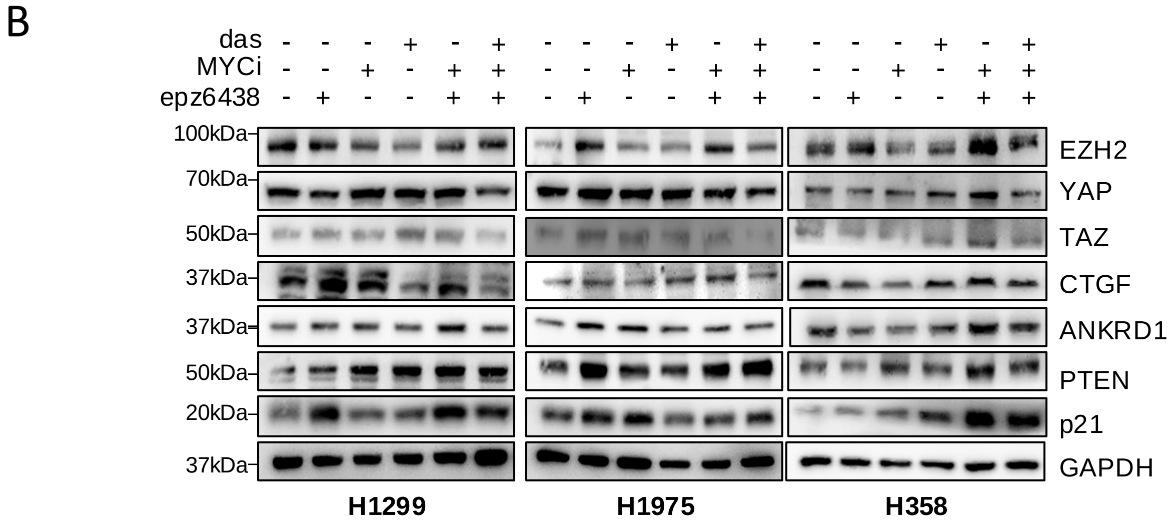

B
das
MYCi
epz6438
100kDa
70kDa
50kDa
37kDa
37kDa
50kDa
20kDa
37kDa
EZH2
YAP
TAZ
CTGF
ANKRD1
PTEN
p21
GAPDH
H1299
H1975
H358

Fig. S7

A

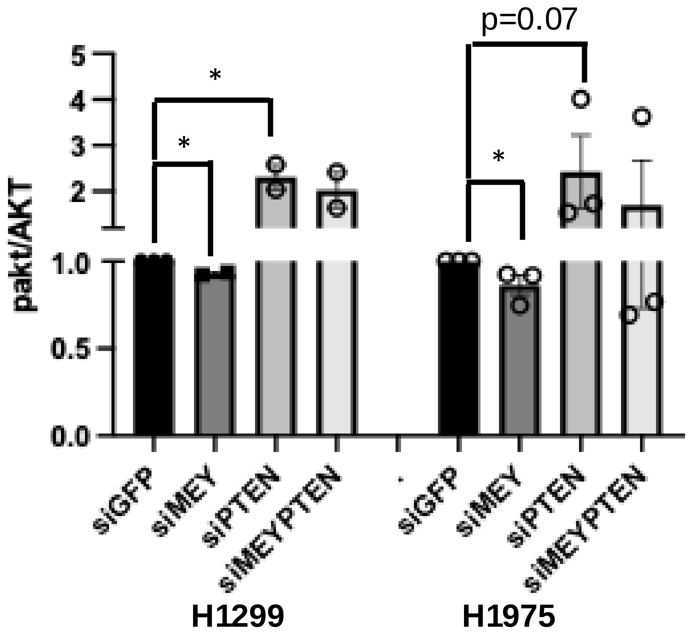

p=0.07
*
*
*
pakt/AKT
5
4
3
2
1.0
0.5
0.0
siGFP
siMEY
siPTEN
siMEYPTEN
siGFP
siMEY
siPTEN
siMEYPTEN
H1299
H1975

B

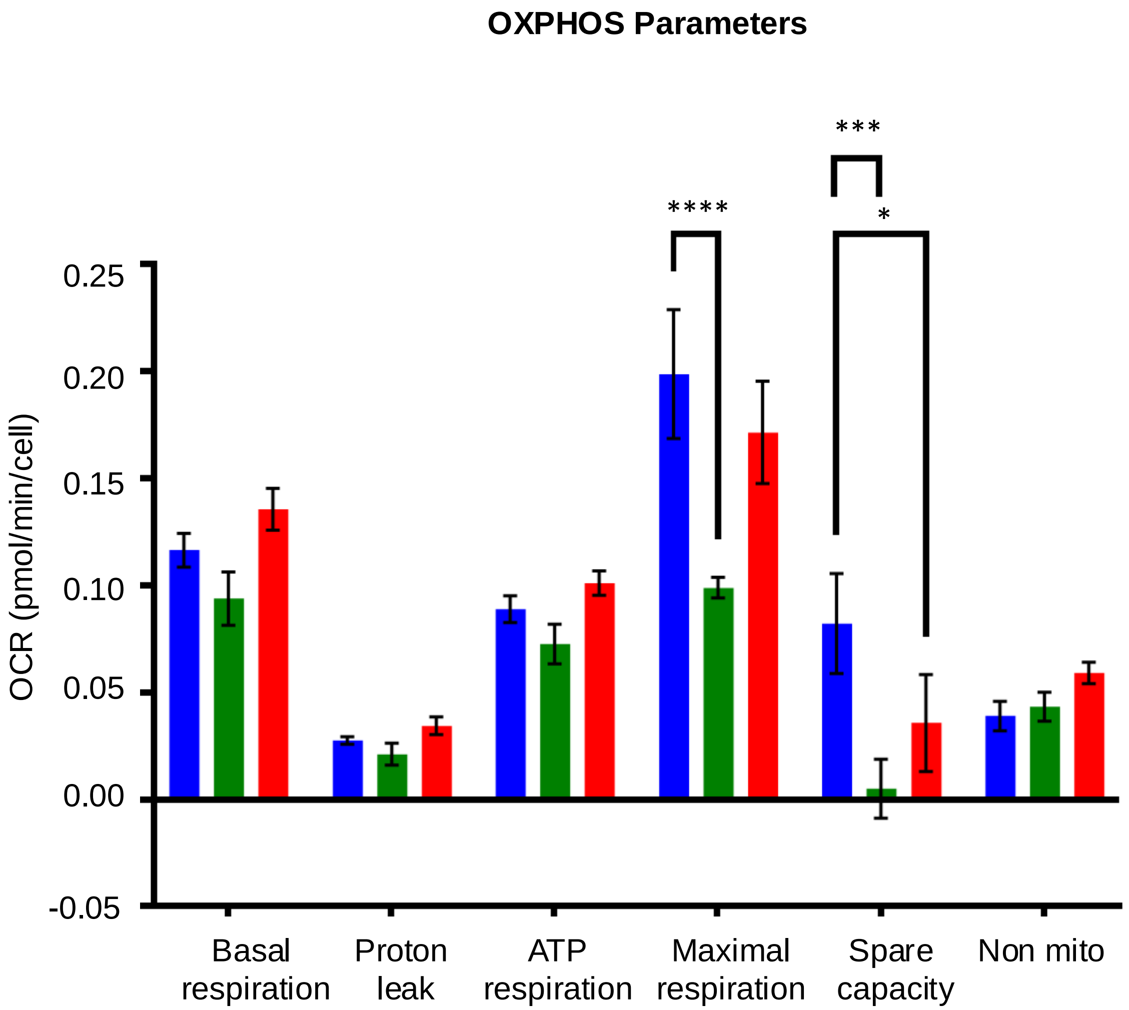

OXPHOS Parameters
***
****
*
OCR (pmol/min/cell)
0.25
0.20
0.15
0.10
0.05
0.00
-0.05
Basal respiration
Proton leak
ATP respiration
Maximal respiration
Spare capacity
Non mito

Fig. S8

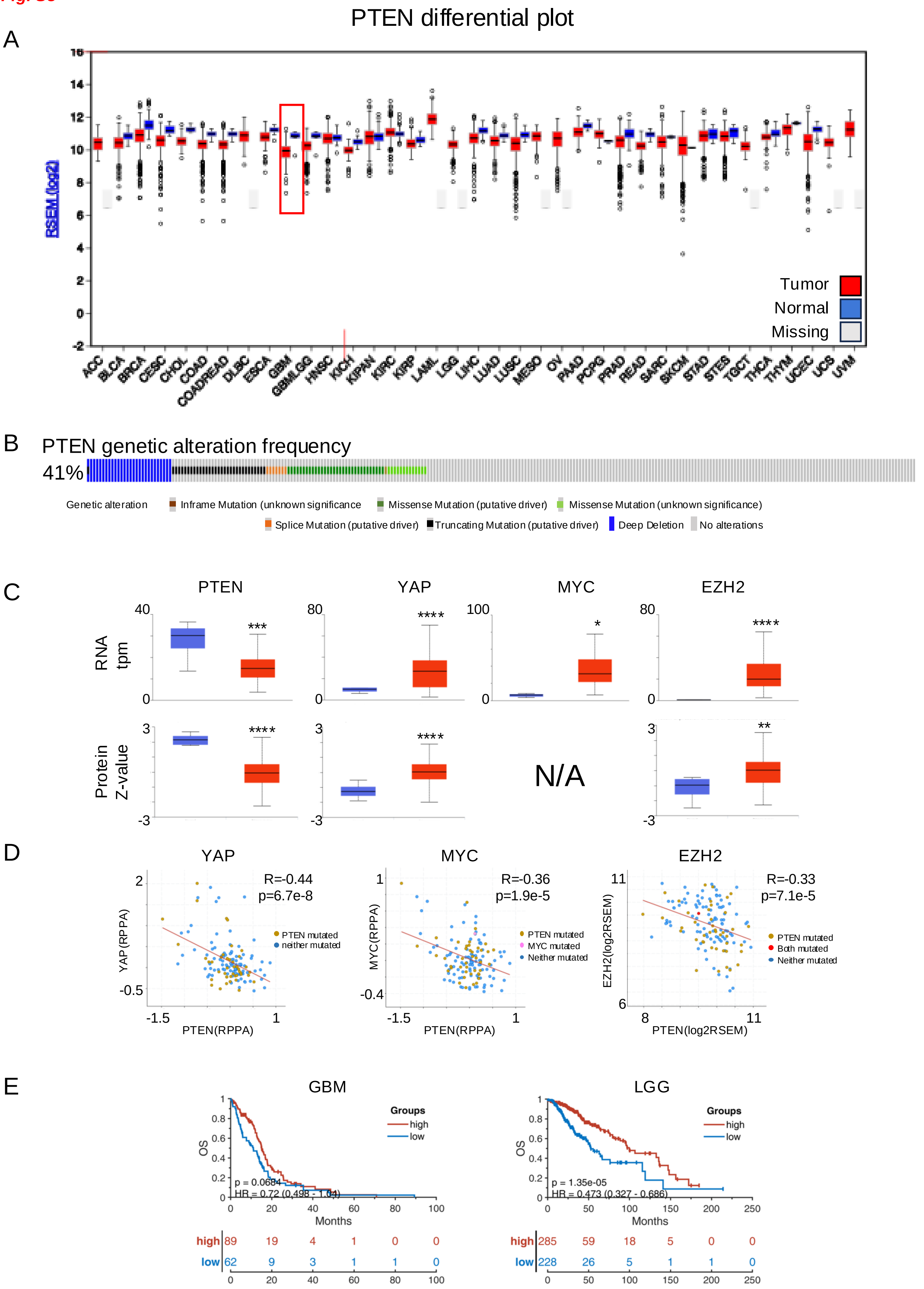

PTEN differential plot
A
RSEM (log2)
Tumor
Normal
Missing
ACC BLCA BRCA CESC CHOL COAD COADREAD DLBC ESCA GBM GBMLGG HNSC KICH KIPAN KIRC KIRP LAML LGG LIHC LUAD LUSC MESO OV PAAD PCPG PRAD READ SARC SKCM STAD STES TGCT THCA THYM UCEC UCS UVM
B
PTEN genetic alteration frequency
41%
Genetic alteration
Inframe Mutation (unknown significance
Missense Mutation (putative driver)
Missense Mutation (unknown significance)
Splice Mutation (putative driver)
Truncating Mutation (putative driver)
Deep Deletion
No alterations
C
PTEN
YAP
MYC
EZH2
RNA tpm
Protein Z-value
N/A
D
YAP
R=-0.44
p=6.7e-8
PTEN mutated
neither mutated
YAP (RPPA)
PTEN(RPPA)
MYC
R=-0.36
p=1.9e-5
PTEN mutated
MYC mutated
Neither mutated
MYC(RPPA)
EZH2
R=-0.33
p=7.1e-5
PTEN mutated
Both mutated
Neither mutated
EZH2(log2RSEM)
PTEN(log2RSEM)
E
GBM
Groups
high
low
OS
p = 0.0684
HR = 0.72 (0.498 - 1.04)
Months
high 89 19 4 1 0 0
low 62 9 3 1 1 0
LGG
p = 1.35e-05
HR = 0.473 (0.327 - 0.686)
high 285 59 18 5 0 0
low 228 26 5 1 1 0

Fig. S9 (PANEL 1C)

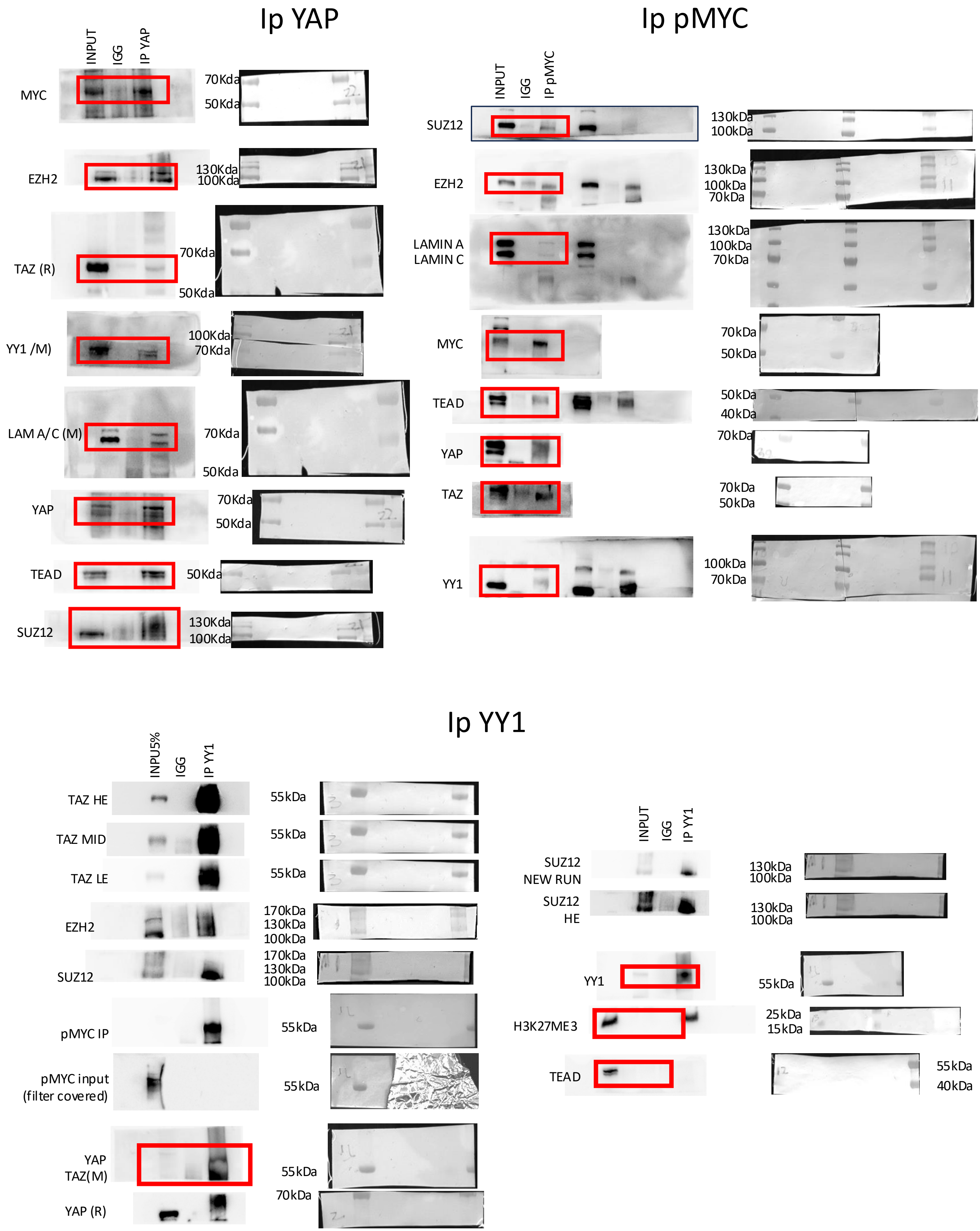

Fig. S10

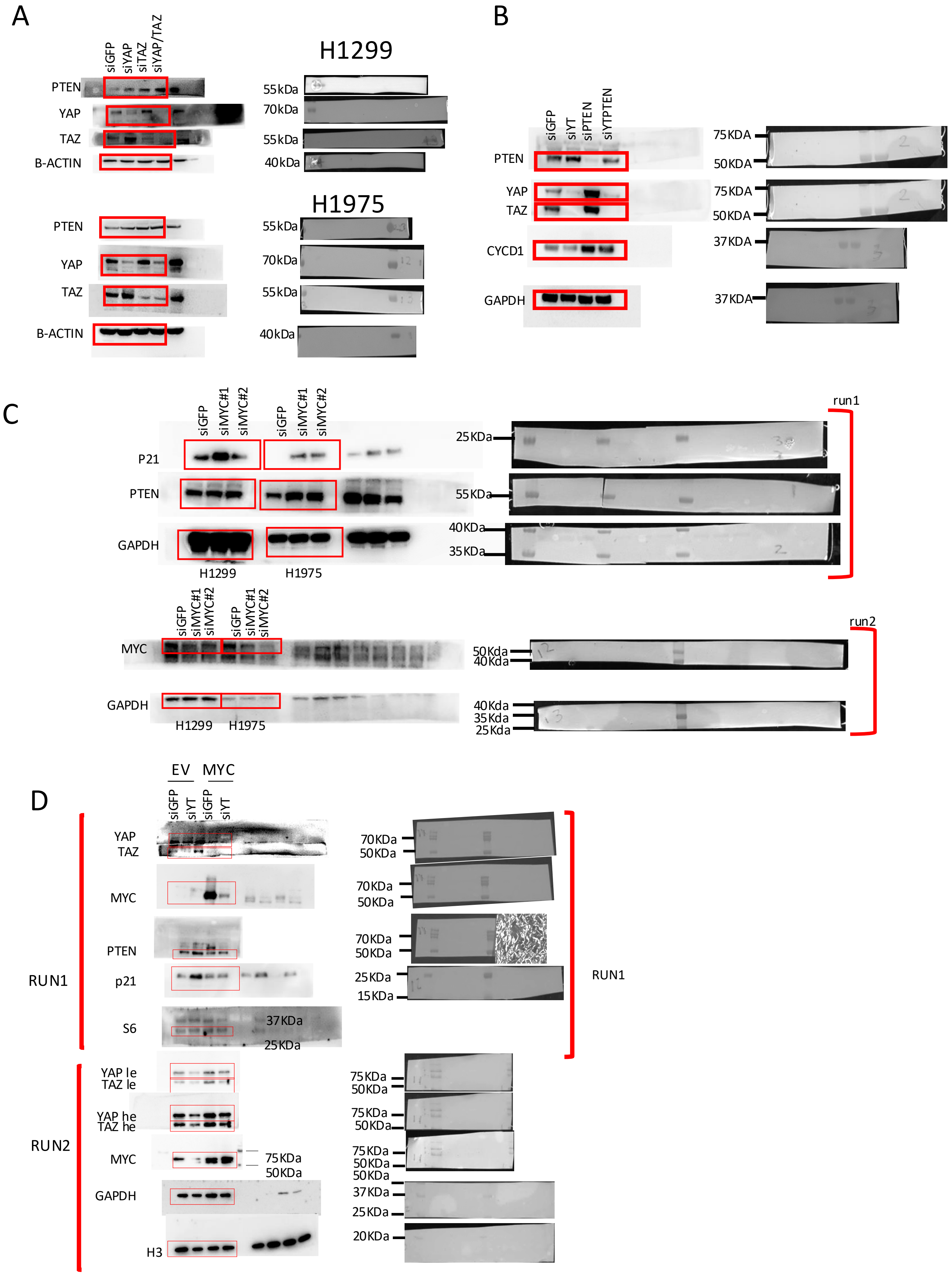
A
siGFP
siYAP
siTAZ
siYAP/TAZ
PTEN
YAP
TAZ
B-ACTIN
H1299
55kDa
70kDa
55kDa
40kDa
H1975
PTEN
YAP
TAZ
B-ACTIN
55kDa
70kDa
55kDa
40kDa
B
siGFP
siYT
siPTEN
siYTPTEN
PTEN
YAP
TAZ
CYCD1
GAPDH
75KDA
50KDA
75KDA
50KDA
37KDA
37KDA
C
siGFP
siMYC#1
siMYC#2
siGFP
siMYC#1
siMYC#2
P21
PTEN
GAPDH
H1299
H1975
25KDa
55KDa
40KDa
35KDa
run1
siGFP
siMYC#1
siMYC#2
siGFP
siMYC#1
siMYC#2
MYC
GAPDH
H1299
H1975
50Kda
40Kda
40Kda
35Kda
25Kda
run2
D
EV
MYC
siGFP
siYT
siGFP
siYT
RUN1
YAP
TAZ
MYC
PTEN
p21
S6
37KDa
25KDa
70KDa
50KDa
70KDa
50KDa
70KDa
50KDa
25KDa
15KDa
RUN1
RUN2
YAP le
TAZ le
YAP he
TAZ he
MYC
GAPDH
H3
75KDa
50KDa
75KDa
50KDa
75KDa
50KDa
75KDa
50KDa
50KDa
37KDa
25KDa
20KDa

Fig. S11

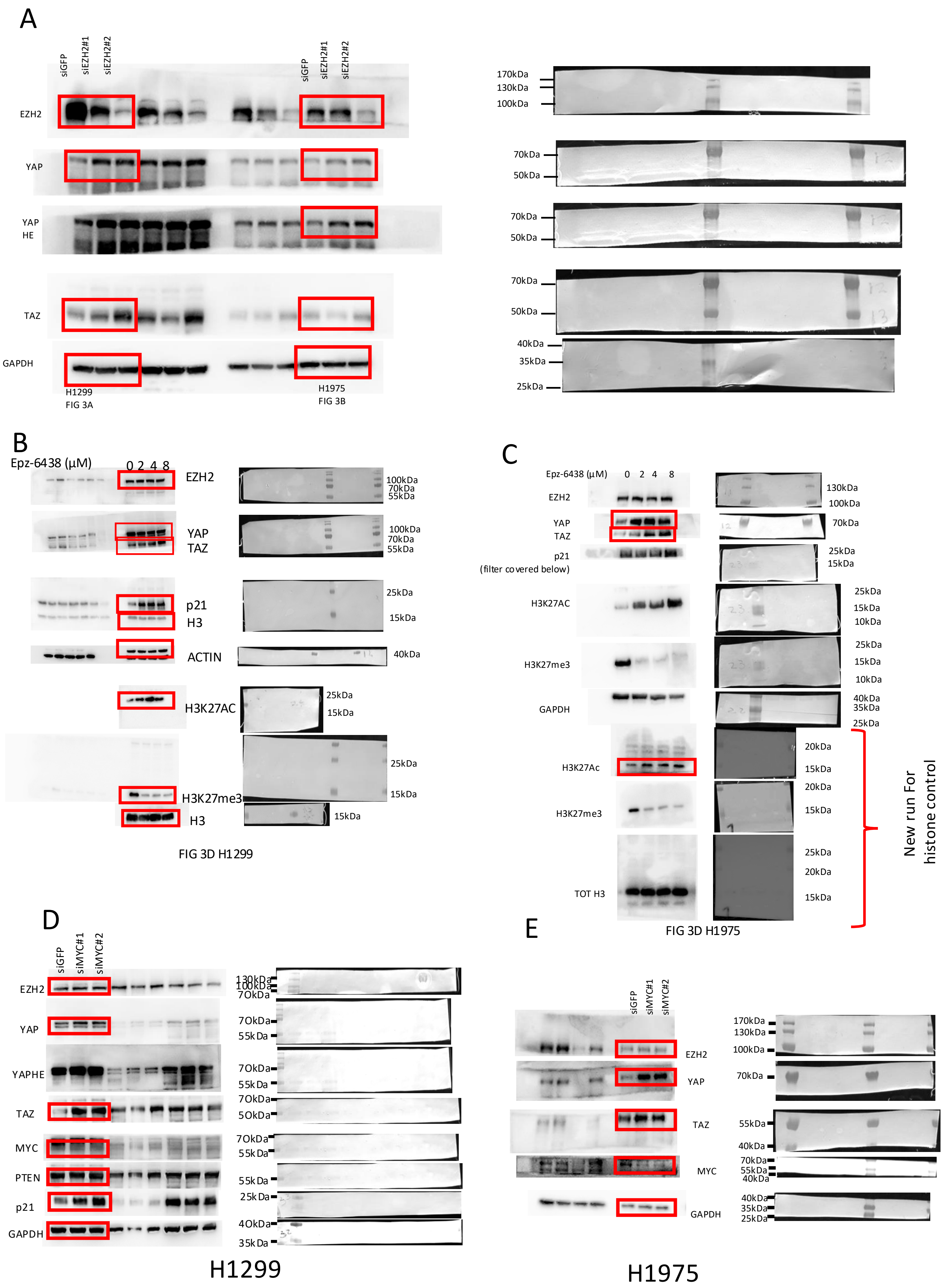
A
siGFP
siEZH2#1
siEZH2#2
siGFP
siEZH2#1
siEZH2#2
EZH2
YAP
YAP HE
TAZ
GAPDH
H1299 FIG 3A
H1975 FIG 3B
170kDa
130kDa
100kDa
70kDa
50kDa
70kDa
50kDa
70kDa
50kDa
40kDa
35kDa
25kDa
B
Epz-6438 (µM)
0 2 4 8
EZH2
100kDa 70kDa 55kDa
YAP TAZ
100kDa 70kDa 55kDa
p21 H3
25kDa
15kDa
ACTIN
40kDa
H3K27AC
25kDa
15kDa
H3K27me3
25kDa
15kDa
H3
15kDa
FIG 3D H1299
C
Epz-6438 (µM) 0 2 4 8
EZH2
130kDa
100kDa
YAP
TAZ
70kDa
p21
(filter covered below)
25kDa
15kDa
H3K27AC
25kDa
15kDa
10kDa
H3K27me3
25kDa
15kDa
10kDa
GAPDH
40kDa
35kDa
25kDa
H3K27Ac
20kDa
15kDa
H3K27me3
20kDa
15kDa
TOT H3
25kDa
20kDa
15kDa
New run For histone control
FIG 3D H1975
D
siGFP
siMYC#1
siMYC#2
EZH2
YAP
YAPHE
TAZ
MYC
PTEN
p21
GAPDH
130kDa
100kDa
70kDa
70kDa
55kDa
70kDa
55kDa
70kDa
50kDa
70kDa
55kDa
55kDa
25kDa
40kDa
35kDa
H1299
E
siGFP
siMYC#1
siMYC#2
EZH2
YAP
TAZ
MYC
GAPDH
170kDa
130kDa
100kDa
70kDa
55kDa
40kDa
70kDa
55kDa
40kDa
40kDa
35kDa
25kDa
H1975

Fig. S12

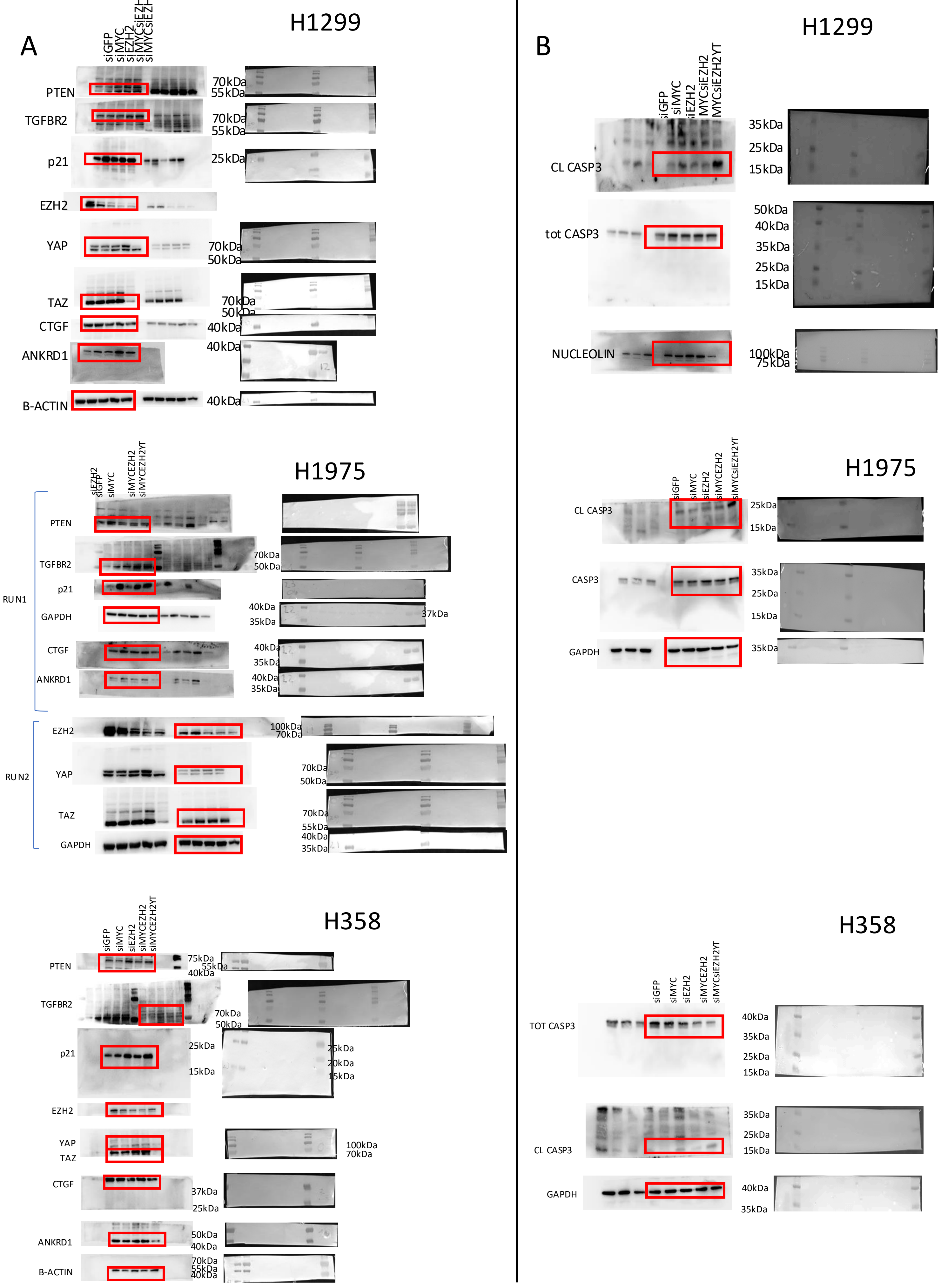
A
H1299
siGFP
siMYC
siEZH2
siMYCsiEZH2
siMYCsiEZH2YT
PTEN
TGFBR2
p21
EZH2
YAP
TAZ
CTGF
ANKRD1
B-ACTIN
70kDa
55kDa
25kDa
50kDa
40kDa
H1975
siEZH2
siGFP
siMYC
siMYCEZH2
siMYCEZH2YT
RUN1
RUN2
GAPDH
37kDa
35kDa
100kDa
H358
siMYCEZH2YT
75kDa
20kDa
15kDa
B
CL CASP3
tot CASP3
NUCLEOLIN
35kDa
CASP3
TOT CASP3
siMYCsiEZH2YT

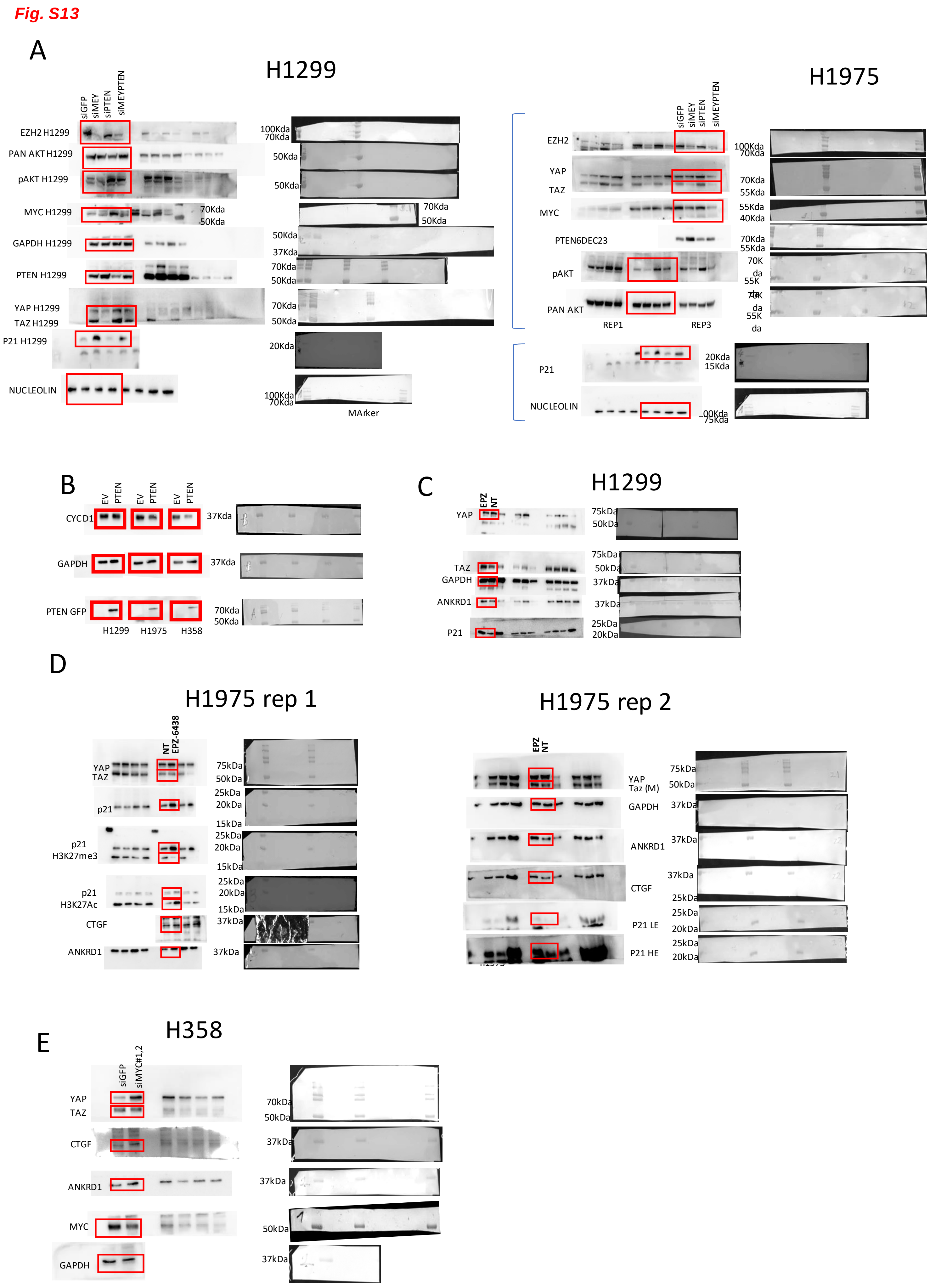
Fig. S13
A
H1299
siGFP
siMEY
siPTEN
siMEYPTEN
EZH2 H1299
PAN AKT H1299
pAKT H1299
MYC H1299
GAPDH H1299
PTEN H1299
YAP H1299
TAZ H1299
P21 H1299
NUCLEOLIN
100Kda
70Kda
50Kda
37Kda
20Kda
MArker
H1975
EZH2
YAP
TAZ
MYC
PTEN6DEC23
pAKT
PAN AKT
REP1
REP3
P21
15Kda
55Kda
40Kda
B
EV
PTEN
CYCD1
GAPDH
PTEN GFP
H1299
H1975
H358
C
EPZ
NT
ANKRD1
75kDa
50kDa
37kDa
25kDa
20kDa
D
H1975 rep 1
EPZ-6438
p21
H3K27me3
H3K27Ac
CTGF
15kDa
H1975 rep 2
Taz (M)
P21 LE
P21 HE
E
H358
siMYC#1,2
70kDa

Fig. S14

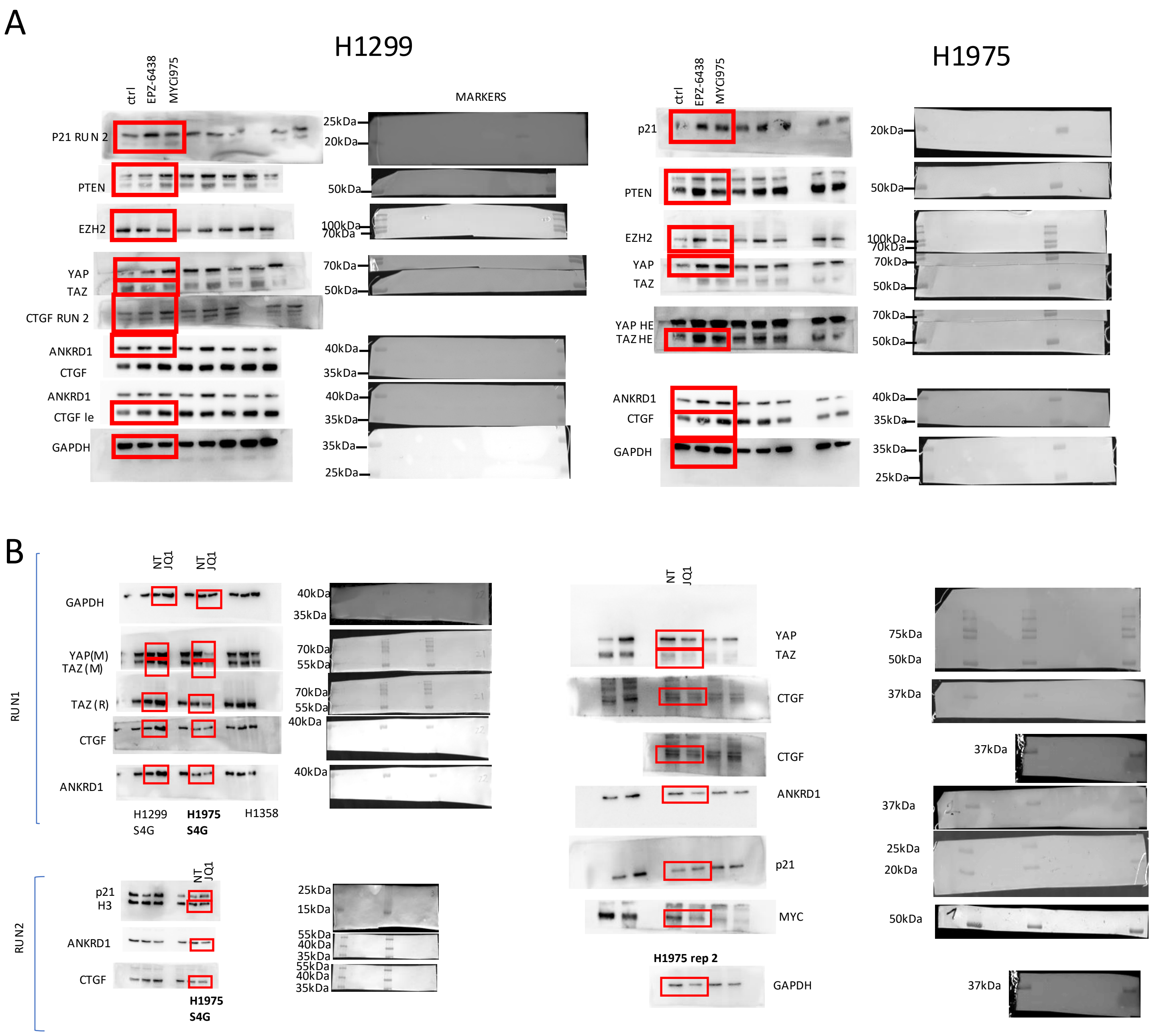
A
H1299
ctrl
EPZ-6438
MYCi975
MARKERS
P21 RUN 2
PTEN
EZH2
YAP
TAZ
CTGF RUN 2
ANKRD1
CTGF
ANKRD1
CTGF le
GAPDH
25kDa
20kDa
50kDa
100kDa
70kDa
70kDa
50kDa
40kDa
35kDa
40kDa
35kDa
35kDa
25kDa
H1975
p21
PTEN
EZH2
YAP
TAZ
YAP HE
TAZ HE
ANKRD1
CTGF
GAPDH
20kDa
50kDa
100kDa
70kDa
70kDa
50kDa
70kDa
50kDa
40kDa
35kDa
35kDa
25kDa
B
RUN1
NT
JQ1
NT
JQ1
GAPDH
YAP(M)
TAZ(M)
TAZ(R)
CTGF
ANKRD1
40kDa
35kDa
70kDa
55kDa
70kDa
55kDa
40kDa
40kDa
H1299
S4G
H1975
S4G
H1358
RUN2
NT
JQ1
p21
H3
ANKRD1
CTGF
25kDa
15kDa
55kDa
40kDa
35kDa
55kDa
40kDa
35kDa
H1975
S4G
NT
JQ1
YAP
TAZ
CTGF
CTGF
ANKRD1
p21
MYC
H1975 rep 2
GAPDH
75kDa
50kDa
37kDa
37kDa
37kDa
25kDa
20kDa
50kDa
37kDa

Fig. S15

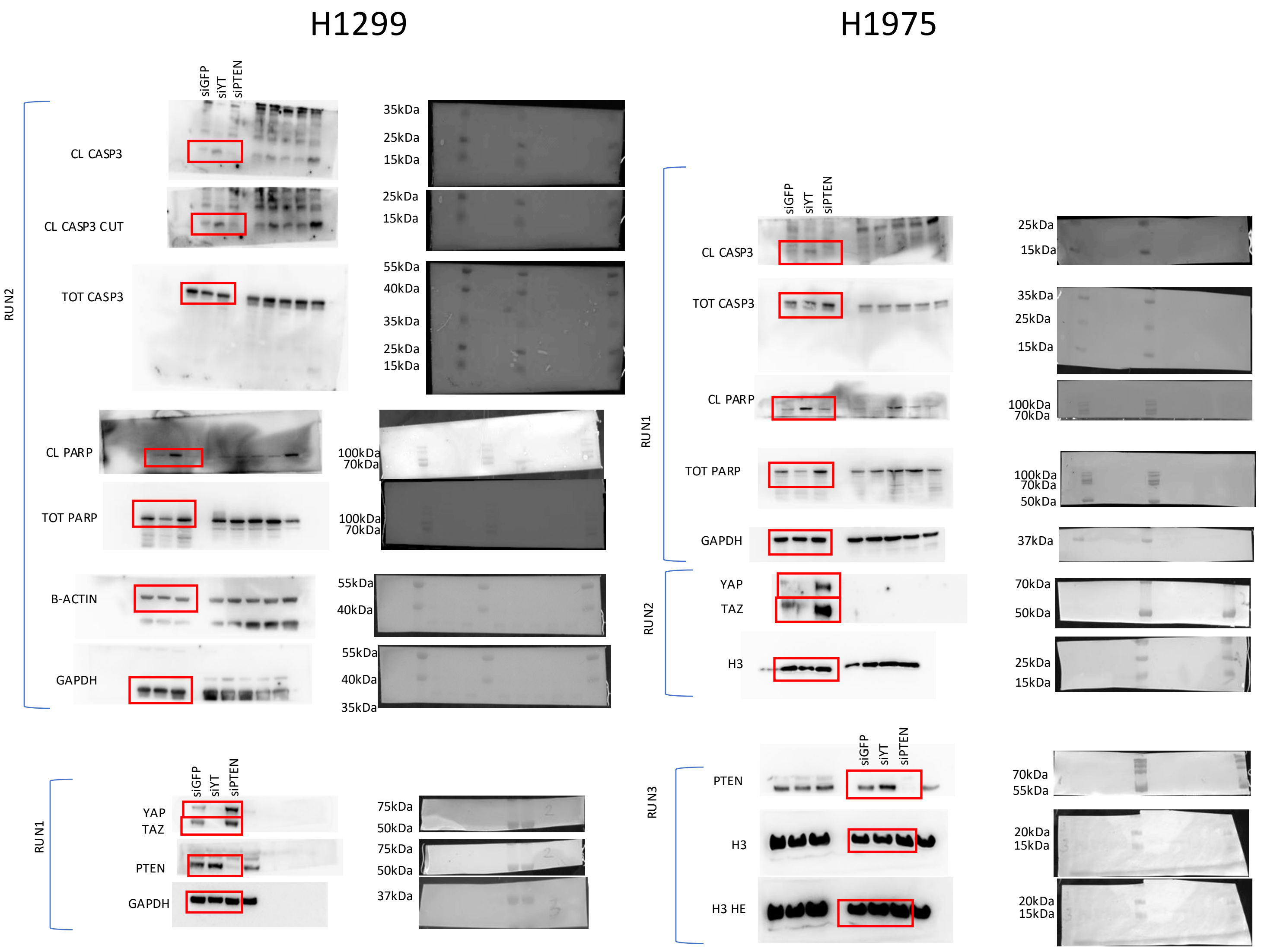
H1299
H1975
siGFP
siYT
siPTEN
RUN2
CL CASP3
CL CASP3 CUT
TOT CASP3
CL PARP
TOT PARP
B-ACTIN
GAPDH
35kDa
25kDa
15kDa
55kDa
40kDa
100kDa
70kDa
RUN1
YAP
TAZ
PTEN
75kDa
50kDa
37kDa
H3
RUN3
H3 HE
20kDa
50kDa

Fig. S16

H1299

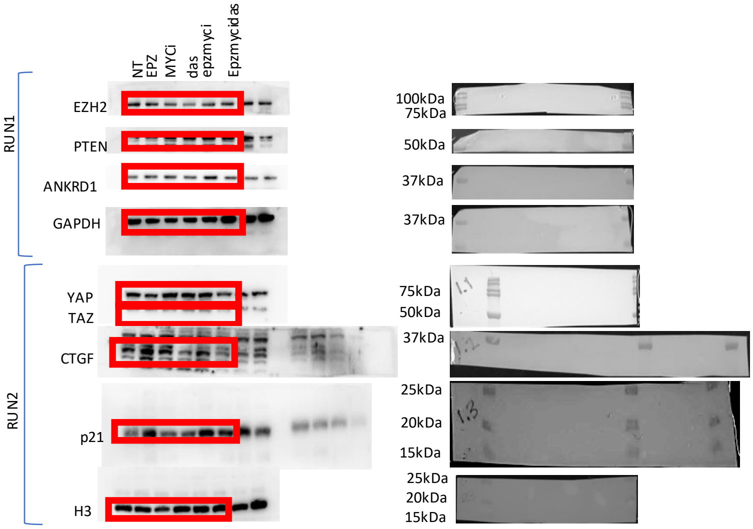

H1975

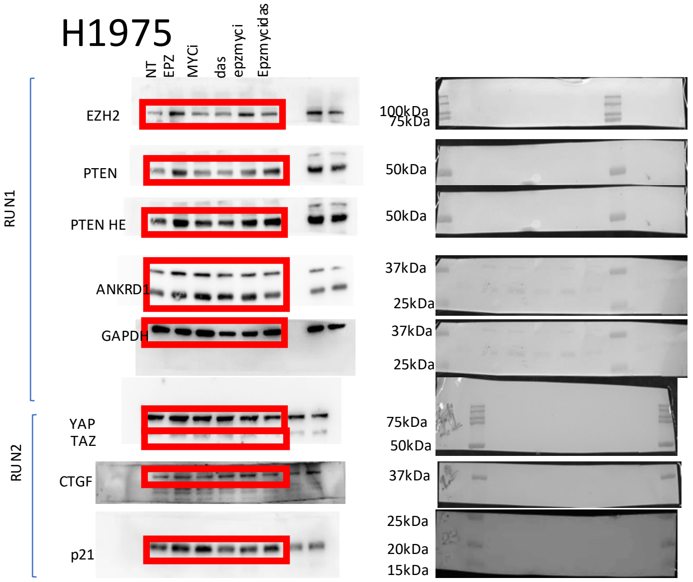

H358

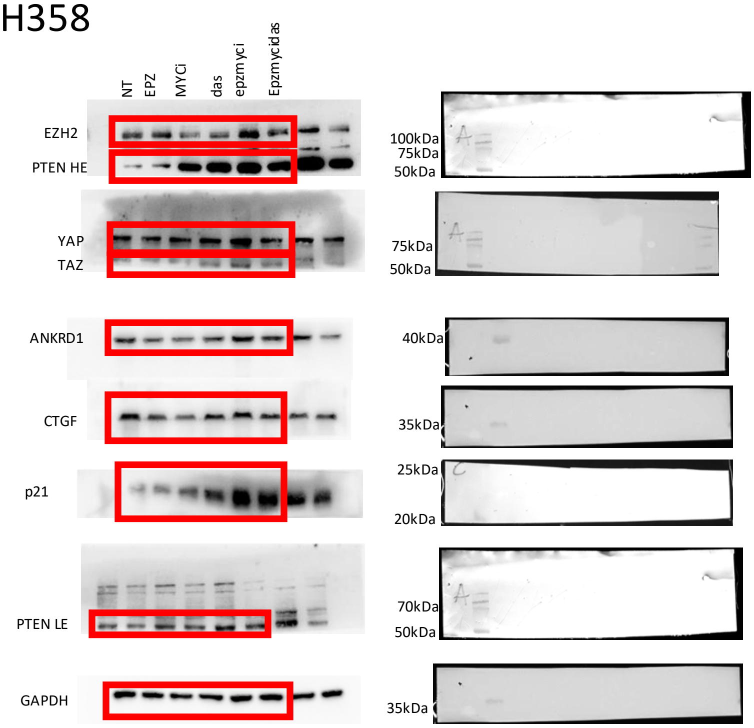

Supplement: Supplementary file 1 — SUPPLEMENTARY FIGURES [file 41420_2024_2216_MOESM1_ESM.pdf]
