# Supplementary material for: The oncogenic axis YAP/MYC/EZH2 impairs PTEN tumor suppression activity enhancing lung tumorigenicity
Source: Cell Death Discov. 2024 Oct 25;10:452. doi: 10.1038/s41420-024-02216-8 (PMC11511861; doi:10.1038/s41420-024-02216-8)
Supplement: Supplementary file 4 — SUPPLEMENTARY MATERIAL [file 41420_2024_2216_MOESM4_ESM.docx]

**Supplementary Figure Legends**

**Fig. S1 (related to Fig. 1):** Immunofluorescence analysis of the indicated proteins in H1975 cells as obtained with Opera Phenix technology. Scale bar of 100 μm is indicated. Arrows indicate example of co-localizations between proteins.

**Fig. S2 (related to Fig. 1):** **A** Hierarchical clustering of significantly upregulated genes upon interference of YAP (left) or TAZ (right) (GSE151200) matched with genes bound by MYC (Chip-Atlas) and also with genes bound by either EZH2 (28) or H3K27me3 (GSE29611) in A549 cells. Clustering was performed basing on Euclidian distance and average linkage method. **B** Venn diagram of the genes negatively regulated by YAP and TAZ and bound by MYC (Chip-Atlas) and EZH2 (28) or H3K27me3 (GSE29611) in A549 cells. **C** Gene set enrichment analysis of genes shown in the heatmaps and listed in Table S1. **D** Cell cycle profile (left) and cell cycle percentage in G1, S, and G2 phases (right) upon PTEN overexpression in H1299, H1975 and H358 cells. **E** WB of the indicated proteins in H1299, H1975 and H358 with or without PTEN overexpression.

**Fig. S3 (related to Fig. 2): A** Boxplots showing differential expression of the indicated transcripts and proteins in LUAD patients as obtained from the UALCAN database, comparing tumoral vs non-tumoral tissues between patients. For differential expression of transcripts, the number of patients analyzed is the following: normal n=59; primary tumor n=515. For differential expression of proteins, the number of patients analyzed is the following: normal n=111; primary tumor n=111. p-values (tumor vs normal) are indicated below: EZH2 protein: p=9.25e-28, EZH2 transcript: p<1e-12. MYC protein: p=1.29e-01, MYC transcript: p=1.58e-01. YY1 protein: p=5.97e-12, YY1 transcript: p<1e-12. PTEN protein: p=2.35E-04, PTEN transcript: p=1.78E-07. Protein levels are measured as Z-value, while transcripts are measured as tpm (transcripts per million). Z-values represent standard deviations from the median across samples. Log2 Spectral count ratio values from CPTAC were first normalized within each sample profile, then normalized across samples. **B** Boxplots showing differential expression of PTEN protein in normal tissue (blue) compared to cancer tissue where Hippo pathway (left panel) or MYC/MYCN pathway (right panel) are altered (red) or not altered (orange). Number of samples are indicated below the boxplots. p-value in left panel: normal vs Hippo pathway altered: p=2.81e-5. Normal vs others: p=2.13e-01. Hippo pathway altered vs others p=1.91e-03. p-value in the right panel: normal vs MYC/MYCN altered: p=7.73e-05. Normal vs others: p=1.9e-01. MYC/MYCN altered vs others: p=2.54e-02. **C** Scatter plot analyses showing the correlation between PTEN protein or transcript with the indicated proteins (RPPA, n=365) or transcripts (RNA Seq V2 RSEM, n=517) in lung adenocarcinoma patients as obtained from the cBioportal database by querying the lung adenocarcinoma TCGA, Firehose Legacy study. R indicates the Pearson’s correlation coefficient. **D** Kaplan Mayer (KM) survival curves, as obtained from the KM plotter tool, of LUAD patients, stratified for tumor stage, with high or low expression of PTEN (Affimetrix ID: 225363_at). The logrank test was used for statistical analysis. Patients were split based on median value.

**Fig. S4 (related to Fig. 3)**: **A** Representative image (top) and quantification (bottom) of colony forming potential in H1299, H1975 and H358 with and without the indicated doses of EPZ-6438. Charts represent mean and SEM of technical replicates of one representative experiment that was repeated three times. Two-tailed t-test was applied to calculate the p-value. H1299 ctrl vs 2μM EPZ-6438 p=0.01, n=3. ctrl vs 4μM EPZ-6438 p=0.007, n=3. ctrl vs 2μM EPZ-6438 p=0.004, n=3. **B** WB of the indicated protein in H1299 and H1975 cells with or without 1μM EPZ-6438. **C** Representative image (top) and quantification (bottom) of colony forming potential in H1975 treated with siYAP/TAZ with or without the indicated doses of EPZ-6438. Charts are relative to a representative biological replicate. H1975 ctrl vs 62.5nM EPZ-6438 p=0.009, n=2. ctrl vs 125nM EPZ-6438 p=0.16, n=2. ctrl vs 250nM EPZ-6438 p=0.04, n=2. **D** WB of the indicated proteins in H358 upon MYC interference with a mix of two alternative siRNAs. **E** WB of the indicated proteins and transcripts in H1299 (left) and H1975 cell line (right) with or without treatment with 250nM tazemetostat or 25nM MYCi975 for 72h. **F** Representative image (top) and quantification (bottom) of colony forming potential in H1299, H1975 and H358 with or without the indicated doses of MYCi975. Charts are relative to a representative biological replicate. The experiments were performed twice for each cell line. **G** WB and real time quantification of the indicated protein and transcripts in H1299 (left) and H1975 cells (right) with or without treatment with 250nM JQ1 for six days. For real time analysis, charts represent mean and SEM of at least three biological replicates. p and n values are indicated below. H1299: ANKRD1 JQ1 treated/untreated: p=0.03 n=7. H1975: ANKRD1 JQ1 treated/untreated: p<0.0001, n=7.

**Fig. S5 (related to Fig. 4)**: **A, B** Scatter plot (top) and quantification (bottom) of the apoptotic rate, as measured through annexinV/PI staining, in H1299 and H1975 cell with or without YAP/TAZ interference (A) or PTEN interference (B) at the indicated time points. Charts indicate mean and SD of technical replicates from a representative biological replicate. The experiments were performed twice for each cell line. Two-tailed t-test was applied to calculate the p-value. H1299 siYT vs siGFP p=0.0005, n=2. H1975 siYT vs siGFP p=0.0004, n=2. H358 siYT vs siGFP p=0.03, n=2. H1299 siPTEN vs siGFP p=0.001, n=2. H1975 siPTEN vs siGFP p=0.0001, n=2. H358 siPTEN vs siGFP p=0.3, n=2. **C** WB analysis of the indicated proteins with or without YAP/TAZ or PTEN interference in H1299 (left) and H1975 cells (right). Numbers above cleaved casp3 and cleaved PARP measure the signal ratio of cleaved over total casp3 or total PARP, respectively. **D** Representative images (top) and relative quantification (bottom) of colony formation in H1299, H1975 and H358 cells at the indicated interference conditions. Charts indicate mean and SEM of three technical replicates from a representative biological replicate. The experiments were performed at least twice for each cell line. Two-tailed t-test was applied to calculate the p-value. H1299 siPTEN vs siGFP p=0.04, n=3. H1975 siPTEN vs siGFP p=0.02, n=3. H1358 siPTEN vs siGFP p=0.01, n=3.

**Fig. S6 (related to Fig. 4): A** Scatter plot (top) and quantification (bottom) of the apoptotic rate, as measured through annexinV/PI staining, in H1299 and H1975 and H358 cells with or without treatment with 250nM Tazemetostat, 25nM MYCi975 and 40nM dasatinib for 72h, either as single or combined treatments. Charts indicate mean and SEM of three technical replicates from a representative biological replicate. The experiments were performed at least twice for each cell line. Two-tailed t-test was applied to calculate the p-value. H1299 MYCi vs ctrl p=0.0006 n=2. EPZ-6438/MYCi/das vs ctrl p=0.04, n=2. H1975 MYCi vs ctrl p=0.03 n=2. EPZ-6438/MYCi/das vs ctrl p=0.01, n=2. H358 EPZ-6438/MYCi/das vs ctrl p=0.08, n=2. **B** WB of the indicated proteins in H1299, H1975 and H358 cells upon treatment with 250nM Tazemetostat, 25nM MYCi975 and 40nM dasatinib for 72h either alone or in combination.

**Fig. S7 (related to Fig. 5): A** Quantification of pAKT signal normalized to total AKT in H1299 and H1975 cells at the indicated interference conditions. Charts represent the average and SEM of two or three independent biological replicates. One-tailed t-test was applied to calculate the p values. P and n values: H1299 pAkt/AKT in siMEY vs siGFP, p=0.005 n=2. siPTEN vs siGFP p=0.002 n=2. H1975 pAkt/AKT in siMEY vs siGFP, p=0.03, n=3. siPTEN vs siGFP, p=0.07, n=3. **B** Mito Stress Test performed in H1299 cells using the Seahorse HS Mini Analyzer. Oxygen Consumption Rate (OCR) values were normalized on the number of cells used in each experimental condition and then used to calculate the following cellular respiratory parameters: basal respiration, proton leak, ATP-linked respiration, maximal respiration, spare capacity and non-mitochondrial respiration. The graph is obtained as a merge of 3 biological replicates. Error bars represent SEM. Two-way Anova was applied to calculate the p-values. Maximal respiration: siGFP vs siMEY p<0.0001, n=3. Spare capacity siGFP vs siMEY p=0.0006, n=3.

**Fig. S8. A** Boxplots of PTEN expression profile in different cancer types from patient samples deposited in the TCGA casuistry as obtained from the FireBrowse Gene Expression Viewer (http//firebrowse.org). GMB is highlighted with a red box. **B** Representation of the frequency of PTEN genomic alterations in 273 patient samples with mutation and CNA (Copy Number Alteration) data from the Glioblastoma Multiforme (TCGA, Firehose Legacy) cohort. **C** Boxplots showing differential expression of the indicated transcripts and proteins in Glioblastoma Multiforme (GBM) patients as obtained from the UALCAN database, comparing tumoral vs non-tumoral tissues between patients. For differential expression of transcripts, the number of patients analyzed is the following: normal n=5; primary tumor n=156 (TCGA samples). For differential expression of proteins, the number of patients analyzed is the following: normal n=10; primary tumor n=99 (CPTAC samples). p-values are indicated below: PTEN transcript: p=3e-04. YAP transcript p=2.2e-08. MYC transcript p= 3.81e-02. EZH2 transcript p <1e-12. PTEN protein p= 1.1e-15. YAP protein p=1.6E-06. EZH2 protein p= 7.8e-04. Data on MYC protein is not available (N/A) in this patient cohort. Protein levels are measured as Z-value, while transcripts are measured as tpm (transcripts per million). Z-values represent standard deviations from the median across samples. Log2 Spectral count ratio values from CPTAC were first normalized within each sample profile, then normalized across samples. **D** Scatter plot analyses showing the correlation between PTEN with the indicated proteins (RPPA, n=138) or transcripts (RNA Seq V2 RSEM, n=136) as obtained from the cBioportal database by querying the Glioblastoma multiforme TCGA, Firehose Legacy study. R indicates the Pearson’s correlation coefficient. For EZH2, protein data was not available in this patient cohort, so we analyzed the correlation between EZH2 and PTEN transcripts. **E** Kaplan-Mayer overall survival curves, as obtained from LGG/GBM TCGA patients, with high or low expression of PTEN transcript (RNAseq). The logrank test was used for statistical analysis. Patients were split based on median value.

**Figs. S9-S16:** Original WB

**Supplementary table S1**: Sheets 1, 2: MYC targets from chip-seq atlas in at least 2 chip-seqs out of 4 in A549 deregulated in RNA-Seq GSE151200 upon siYAP (sheet1) or siTAZ (sheet 2). Column F: Pearson correlation between listed transcripts and MYC expression in NSCLC. Column G: p-value of this correlation. In column H and I the presence of either EZH2 or H3k27me3 is highlighted. Genes with pval<0.05 & padj<0.3 were selected as significative. Sheet 3: columns K, L: genes modulated by YAP or TAZ in RNA-Seq GSE151200 and bound by MYC, EZH2, H3K27me3. Columns A-G: gene set enrichment analysis of HALLMARK pathways for genes listed in columns K, L. Analysis was performed by ShinyGO web tool (http://bioinformatics.sdstate.edu/go/).

**Supplementary table S2:** sheet 1,2: transcripts (sheet 1) and proteins (sheet 2) correlated to PTEN in LUAD patients as obtained from the cBiCoportal database. The data set used for the analysis is the Lung Adenocarcinoma Firehose Legacy. Sheet 3: Gene set enrichment analysis of HALLMARK pathways for proteins anticorrelated to PTEN in LUAD. Analysis was performed by ShinyGO web tool (http://bioinformatics.sdstate.edu/go/).

**Supplementary materials:**

**Primers used for ChIp analysis**:

chIp PTEN p1-F: CAAAGGAAGAAGACGACTTGCCTCC

chIp PTEN p1-R: GGCGAGGAGACCTAGGAGGGT

chIp PTEN p2-F: GGCGGAGGGGGCGGGCA

chIp PTEN p2-R: ATCTCCCTCGCCTGAGCCCG

chIp CTGFp-F: GGAGTGGTGCGAAGAGGATA

chIp CTGFp-R: GCCAATGAGCTGAATGGAGT

chIp ANKRD1p-F: AAAAAGGGCAGTGATGTGGTG

chIp ANKRD1p-R: GAAGAGGGAGGGGAGGACAA

chIp CYR61p-F: CACACACAAAGGTGCAATGGAG

chIp CYR61p-R: CCGGAGCCCGCCTTTTATAC

chIp neg ctrl: FTCCTCTGGCATTTCTAGGGAATGTGGT

chIp neg ctrl: RCAGACGTACTAAAATACAATATAGGAAAAACCAAACT

**Primers used for transcript analysis:**

RT-GAPDH-F: GAGTCAACGGATTTGGTCGT

RT-GAPDH-R: GACAAGCTTCCCGTTCTCAG

RT-YAP-F: CACAGCATGTTCGAGCTCAT

RT-YAP-R: GATGCTGAGCTGTGGGTGTA

RT-TAZ-F: CCATCACTAATAATAGCTCAGATC

RT-TAZ-R: GTGATTACAGCCAGGTTAGAAAG

RT-EZH2-F: TGGTTAACGGTGATCACAGGA

RT-EZH2-R: GGAGGTAGCAGATGTCAAGG

RT-TGFBR2-F: GTAGCTCTGATGAGTGCAATGAC

RT-TGFBR2-R: CAGATATGGCAACTCCCAGTG

RT-p15-F: GGACTAGTGGAGAAGGTGCG

RT-p15-R: CATCATGACCTGGATCGCGC

RT-p21-F: GGGACAGCAGAGGAAGAC

RT-p21-R: GCGTTTGGAGTGGTAGAAATC

RT-MYC-F: CTCCTGGCAAAAGGTCAGAG

RT-MYC-R: TCGGTTGTTGCTGATCTGTC

RT-PTEN-F: AGTTCCCTCAGCCGTTACCT

RT-PTEN-R: AGGTTTCCTCTGGTCCTGGT

RT-CTGF-F: GCCACAAGCTGTCCAGTCTAATCG

RT-CTGF-R: TGCATTCTCCAGCCATCAAGAGAC

RT-ANKRD1-F: AGTAGAGGAACTGGTCACTGG

RT-ANKRD1-R: TGGGCTAGAAGTGTCTTCAGAT

RT-CYR61-F: CCTTGTGGACAGCCAGTGTA

RT-CYR61-R: ACTTGGGCCGGTATTTCTTC

RT-CYCD1-F: AACTACCTGGACCGCTTCCT

RT-CYCD1-R: TCGGTGTAGATGCACAGCTT

**siRNA used for interference experiments:**

siGFP (as non-silencing control): AAGUUCAGCGUGUCCGGGGAG, siYAP#1: GACAUCUUCUGGUCAGAGA, siYAP#2: CUGGUCAGAGAUACUUCUU, siTAZ#1: AAAGUUCCUAAGUCAACGU, siTAZ#2: AGGUACUUCCUCAAUCACA, siEZH2#1: GCUGACCAUUGGGACAGUATT, siEZH2#2: GUGUAUGAGUUUAGAGUCATT,

siMYC#1: GCCACAGCAUACAUCCUGU, siMYC#2: GGACUAUCCUGCUGCCAAG,

siPTEN: GCUACCUGUUAAAGAAUCA, si YY1#1: CGACGACUACAUUGAACAATT, siYY1#2: GAACUCACCUCCUGAUUAU.

**Antibodies used in this study:**

The following antibodies were used: Rabbit monoclonal anti-YAP (Invitrogen, #PAI46189), mouse monoclonal anti YAP (Santa Cruz, #sc-101199), mouse monoclonal anti-TEF1 (BD-Transduction Laboratories, #610923), rabbit monoclonal anti-p21 Waf1/Cip1 (Cell Signaling, #2947), rabbit polyclonal anti-TGFBR2 (C-16, Santa-Cruz #sc-220, 1:200), rabbit polyclonal anti-PTEN (Cell Signaling, #9552) rabbit polyclonal anti EZH2, (Bethyl Laboratories, #A304-1974), rabbit polyclonal anti-SUZ12 (Bethyl Laboratories, #A302-407A) mouse monoclonal anti-Lamin A/C (Santa Cruz, #sc7292), mouse monoclonal anti-MYC (9E-10, Santa Cruz, #sc-40) rabbit polyclonal anti-YY1 (Invitrogen, #PA5-29171), rabbit monoclonal anti-pMYC (Abcam, #ab185656), mouse monoclonal anti-α-tubulin (Abcam, #ab7291, mouse monoclonal anti nucleolin (Santa Cruz #sc-8031), mouse monoclonal anti-CTGF (Santa Cruz #sc-373936), rabbit polyclonal anti-ANKRD1(Abcam, #ab272894) rabbit monoclonal anti-H3k27me3 (Millipore, #cs200603), rabbit monoclonal anti-H3 (Abcam, #ab1791), mouse monoclonal anti-H3k27Ac (Cell Signaling, #200551), rabbit polyclonal Anti-RNA pol II CTD repeat YSPTSPS (phospho S5) antibody (Abcam, #ab5131), mouse monoclonal anti-CASP3 (Enzo Life Science #31A1067), rabbit polyclonal anti-PARP (Cell Signaling #9542) and rabbit anti-IgG (H-270, Santa Cruz #sc-66931). For western blot, primary antibody was diluted 1:1000 if not differently indicated. Secondary antibodies used for western blot were goat anti-mouse and goat anti-rabbit, conjugated to horseradish peroxidase (Amersham Biosciences, Piscataway, NJ) diluted 1:10000 in TBS with 5%BSA and 0,1% tween. For immunofluorescence and PLA, primary antibodies were used 1:25-1:100 depending on the antibody. Secondary antibodies used for immunofluorescence were Goat anti Rabbit
Alexa Fluor™ 488, Goat anti Mouse Alexa Fluor™ 594 and Goat anti Mouse Alexa Fluor™ 647 and were diluted 1:200.
